# Photodiodes embedded within electronic textiles

**DOI:** 10.1038/s41598-018-34483-8

**Published:** 2018-11-01

**Authors:** Achala Satharasinghe, Theodore Hughes-Riley, Tilak Dias

**Affiliations:** Advanced Textiles Research Group, School of Art & Design, Nottingham Trent University, Bonington Building, Dryden Street, Nottingham, NG1 4GG UK

## Abstract

A novel photodiode-embedded yarn has been presented and characterized for the first time, offering new possibilities for applications including monitoring body vital signs (including heart rate, blood oxygen and skin temperature) and environmental conditions (light, humidity and ultraviolet radiation). To create an E-Textile integrated with electronic devices that is comfortable, conformal, aesthetically pleasing and washable, electronic components are best integrated within the structure of a textile fabric in yarn form. The device is first encapsulated within a protective clear resin micro-pod before being covered in a fibrous sheath. The resin micro-pod and covering fibres have a significant effect on the nature of light received by the photoactive region of the device. This work characterised the effects of both encapsulating photodiodes within resin micro-pods and covering the micro-pod with a fibrous sheath on the opto-electronic parameters. A theoretical model is presented to provide an estimate for these effects and validated experimentally using two photodiode types and a range of different resin micro-pods. This knowledge may have wider applications to other devices with small-scale opto-electronic components. Wash tests confirmed that the yarns could survive multiple machine wash and drying cycles without deterioration in performance.

## Introduction

With the rapid growth of interest in wearable electronics, optical sensors equipped with photodiodes (PDs) have become widely employed in a range of wearable and portable devices for body vitals monitoring^1–4^, proximity sensing^5^ and environmental monitoring^6,7^ applications. Wearable, optical spectroscopy is also an emerging area of research, especially for monitoring concentrations of chromophores in biological tissue^8,9^.

PDs generate a small electrical current and a change in the voltage difference based on the nature of the incident light received at the photoactive region of the device. PDs are extensively used in commercial digital imaging and sensing devices across various fields^10,11^ and their wide-spread employment has resulted in the development of small, low-cost devices.

Despite the capabilities, versatility and potential in wearable applications being well understood, the form factor of photodiode enabled wearable devices are limited to accessories such as wristbands^12^, jewellery^13^ or similar rigid, non-textile based products or hand held portable devices^14^. There were efforts on incorporating similar electronic components onto textiles by superficially attaching them onto the fabric surface. However, the adoption rates of such smart textiles has been slow, mainly owing to lack of normalcy and poor durability^15–18^. A recent work on embedding photodiodes inside optical fibres was presented for fabric based applications^19^.

**Figure 1:**
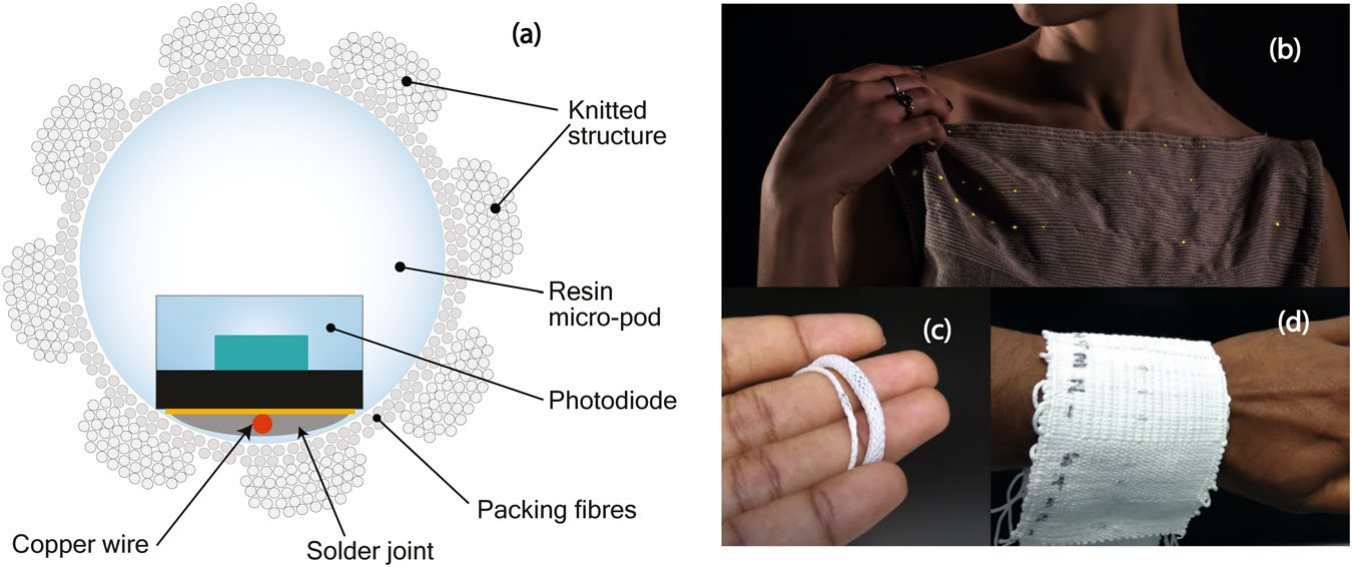
Electronic yarns and resultant E-textiles. **(a)** A schematic of the cross sectional view of photodiode embedded yarn. **(b)** E-textile with LED embedded yarns from pervious developments. **(c)** Photodiode embedded yarns. **(d)** Woven fabric made with photodiode-embedded yarns for wash testing.

This research makes use of electronic yarn (E-yarn) technology^20^ which is a patented platform technology that enables small-scale semiconductor devices to be integrated into the core of textile yarns, making the components completely undetectable to the wearer and washable. This technology has previously been employed for a range of applications^21–23^. Fig. 1(b) shows electronic yarns with integrated LED’s incorporated into a top; the image clearly shows that the resultant woven fabric is flexible and retains its shear characteristics (allowing it to drape).

The E-yarn concept has a particular architecture as depicted in Fig. 1(a). The miniature semiconductor package dice are first soldered onto fine multi-strand copper wires to create robust mechanical and electrical interconnects. The devices are then embedded inside of clear resin micro-pods (RMPs), which protect the embedded devices from external mechanical stresses and chemical agents while also electrically isolating the device from the surrounding. Finally the micro-pod and copper wire are covered by bundles of textile fibres (packing fibres) and small diameter tubular knit-braid structure which provides softness, and a textile like aesthetic and haptic properties^24,25^.

Woven and knitted structures are formed by either interlacing or interloping yarns, and these two techniques of binding yarns together allow yarns and their constituent fibres a high degree of freedom for movement, allowing textile fabrics to shear, which is the key to the superior ability of textiles to drape around 3D objects. Thin polymer films can be flexible but due to lack of shear behavior they cannot be draped around 3D objects without forming creases. The incorporation of an E-yarn, consisting of a photodiode encapsulated within a resin micro-pod and fine copper wire interconnects, into a woven or knitted fabric will not influence the shear behavior of the fabric as the E-yarn would have the same freedom of movement as other yarns in the fabric. In addition, the volume occupied by the micro-pods and copper interconnects are insignificant compared to the total volume of textile fibres in a fabric. This results in conformal, drapable, mechanically robust yarns and fabrics (Fig. 1(b–d)), which can withstand multiple wash cycles.

In this research, the E-yarn technology platform was used to produce E-yarns embedded with miniature PDs for the first time (creating PD-yarns). The produced PD-yarns can be readily integrated into a textile fabric using weaving methods^26,27^ or an inlay embroidery technique^23^, without significantly altering the textile characteristics of the base fabric. The architecture of these PD-yarns makes them washable and re-useable while maintaining their electronic properties, and textile like haptic and aesthetic character; which will make them more desirable over superficially attached wearable devices^24^. The photodiode embedded yarns will have numerous applications including measuring body vitals such as heart rate^28^, skin temperature^29^, blood glucose level^30^, and blood oxygen level^2,13^ or environmental conditions such as radiation exposure^31^, thermal exposure, and humidity^7^. In addition, they can be utilized for proximity sensing and light-based control systems for lighting textiles and safety gear^32^.

Since the electronic behavior of photodiodes are governed by the nature of incident light received by the photo-active material, there exists a need to understand the effects of the RMP and fibrous sheath on the light received by the PD which this paper investigates. This will enable the design of the PD embedded yarns to be optimized.

Experiments were conducted using two types of commercial surface mounted device (SMD) miniature silicon P-I-N type PDs (TEMD7000x1 and VEMD6060x1 from Vishay Intertechnology Inc., Malvern, PA, USA) hereafter referred to as PD1 and PD2 respectively (additional information is provided in supplementary section 1). The effects of RMP size, resin type, position of the PD inside the RMP, and the fibrous sheath on short circuit current (I_SC_) and open-circuit voltage (V_OC_) of PDs in photovoltaic mode (zero bias)^33,34^ were experimentally determined.

Further, a generalized mathematical model was proposed to estimate the effects of the geometry of the cylindrical RMP (selected due to the cylindrical geometry of fibres and yarns), optical properties of the resin and incident lighting conditions on I_SC_ and V_OC_ of the PDs. The model was derived based on geometrical optics^35–37^ and the fundamentals of photovoltaics^38–40^. The generalized model can be extended to incorporate RMPs with non-uniform three-dimensional features (e.g. hemispherical) which is not within the scope of this work. The generalized model was simplified to represent the experimental scenarios and the experimental results were compared against model-estimated results.

Finally, wash durability tests were conducted in yarn and fabric form to confirm their performance under multiple washing and drying cycles.

## Results and Discussion

This section consists of two parts; the development of the mathematical model followed by experimental results. Unless specifically stated, average value of five repeat tests along with standard deviations (depicted by error bars) are presented for each data point throughout this paper.

### Modelling the effect of the micro-pod encapsulation

In order to understand and characterise the effects of the encapsulated PDs a generalized ray tracing mathematical model which predicts the light intensity within the RMP was proposed.

**Figure 2:**
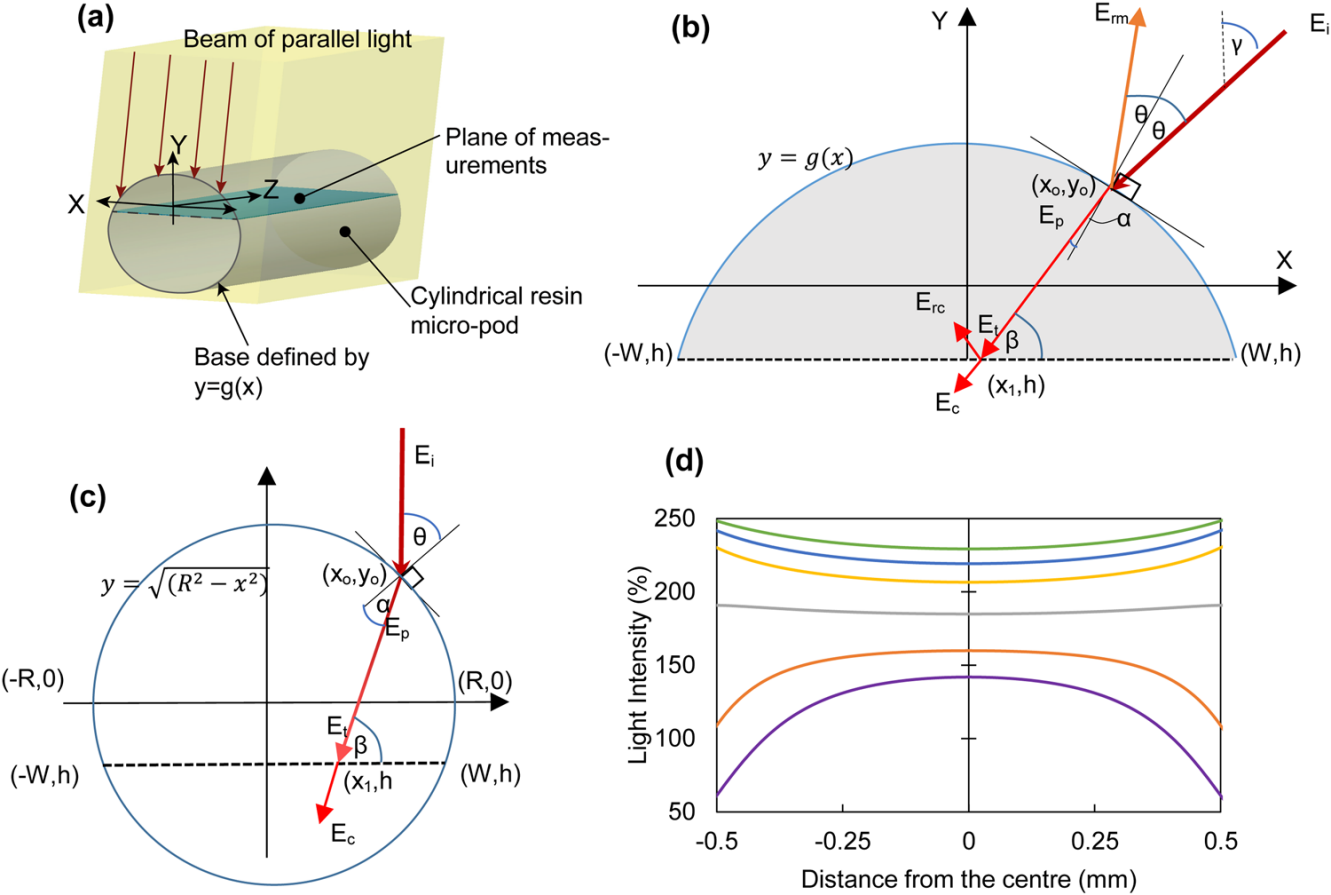
Mathematical model for estimating average light irradiance intensity inside of a cylindrical micro-pod. **(a)** 3D illustration of the micro-pod, plane of measurement and beam of incident light. **(b)** Generalized ray tracing model depicted on a cross sectional view of the cylindrical micro-pod. **(c)** Simplified ray tracing model for circular cross section. **(d)** Estimated intensity variation along the width of the photoactive plane for a TEMD 7000 × 1 photodiode embedded inside 1.5 mm (purple), 1.9 mm (orange), 2.7 mm (grey), 3.8 mm (yellow), 4.8 mm (blue) and 5.8 mm (green) diameter, micro-pods in the standard configuration.

A right-angled cylinder (with its bases given by a function *y* = *g*(*x*)) representing the RMP was defined with respect to a three-axis rectangular co-ordinate system (XYZ) as given in Fig. 2(a). The intersection between the cylindrical RMP and a plane orthogonal to the base of the cylinder (parallel to the XZ plane) was defined as the plane of measurement.

In its simplest terms, a single ray of incident light can be considered, as illustrated in Fig. 2(b). The ray had an intensity *E*_*i*_ and angle γ to the vertical axis, which met the boundary of the cylindrical micro-pod defined by at co-ordinates *x*_0_,*y*_0_. A fraction of the incident ray was reflected (*E*_*r*_) at the boundary surface and the remaining fraction (*E*_*p*_) was refracted into the RMP. The refracted ray was partially absorbed (attenuated) during its travel inside the RMP before reaching the plane of measurement (*E*_*t*_).

Based on the selected geometry, Snell’s law for refraction was used to determine the intersection point of the refracted ray and the photoactive plane as given in Eq. (1).

1 $${x}_{1}=x-[\frac{g(x)-h}{\tan \,\beta }]$$

where β was the angle made by the transmitted ray *E*_*t*_ to the photoactive plane, which was determined by incident angle of the ray (γ), the tangent angle at the boundary surface (*dy*/*dx* at *x*_0_,*y*_0_) and the refractive index of the micro-pod material (*n*_*r*_); *h* was the distance between X-axis and the plane of measurement.

A fraction of the ray (*E*_*rc*_) was partially reflected at the plane of measurement, and the residual ray (*E*_*c*_) was transmitted to the semiconductor.

Based on the geometry and theory of light absorption, the light transmitted to the semiconductor at (*x*_1_,*h*) was given by Eq. (2).

2 $${E}_{c}={E}_{i}\,(1-{k}_{m})(1-{k}_{c})\,{10}^{-{\mu }^{}[g({x}_{o})-h]{\rm{c}}{\rm{o}}{\rm{s}}{\rm{e}}{\rm{c}}\beta }$$

Where μ was the decadic attenuation coefficient for the micro-pod material, *k*_*m*_, and *k*_*c*_ were the fractions of light reflected at the air-micro-pod boundary surface and measurement plane respectively. *k*_*m*_ and *k*_*c*_ were derived using Fresnel equation^36^ as shown below.

31 $${k}_{m}=\frac{1}{2}\{{[\frac{\cos \theta -{n}_{r}\sqrt{1-{(\sin \theta /{n}_{r})}^{2}}}{\cos \theta +{n}_{r}\sqrt{1-{(\sin \theta /{n}_{r})}^{2}}}]}^{2}+{[\frac{{n}_{r}\cos \theta -\sqrt{1-{(\sin \theta /{n}_{r})}^{2}}}{{n}_{r}\cos \theta +\sqrt{1-{(\sin \theta /{n}_{r})}^{2}}}]}^{2}\}$$

32 $${k}_{c}=\frac{1}{2}\{{[\frac{{n}_{r}\sin \beta -{n}_{c}\sqrt{1-{(\frac{{n}_{r}}{{n}_{c}}\cos \beta )}^{2}}}{{n}_{r}\sin \beta +{n}_{c}\sqrt{1-{(\frac{{n}_{r}}{{n}_{c}}\cos \beta )}^{2}}}]}^{2}+{[\frac{{n}_{p}\sin \beta -{n}_{r}\sqrt{1-{(\frac{{n}_{r}}{{n}_{c}}\cos \beta )}^{2}}}{{n}_{c}\sin \beta +{n}_{r}\sqrt{1-{(\frac{{n}_{r}}{{n}_{c}}\cos \beta )}^{2}}}]}^{2}\}$$

Here, *θ* was the angle made by the incident ray to the normal drawn to *y* = *g*(*x*) at *x*_0_,*y*_0_ and *n*_*c*_ was the refractive index of the photoactive surface.

The average light intensity (*E*_*AVG*_) between two points (*x*_*a*_,*h*) and (*x*_*b*_,*h*) on the plane of measurement was given by:

4 $${E}_{AVG}=({\int }_{{x}_{a}}^{{x}_{b}}{E}_{c}.dx)/({x}_{b}-{x}_{a})$$

For a photodiode with a rectangular photo-active area embedded inside of a RMP, and photoactive width matching the measurement plane discussed in the above ray tracing model, the irradiance intensity on the photodiode *E* (synonymous to the term *E*_*AVG*_ used in the ray tracing model) can be estimated.

The relationships between short-circuit current and irradiance intensity is linear for a photocell^41^. Therefore, the short circuit current for an arbitrary irradiance intensity level *E* can be given based on a baseline irradiance intensity *E*_*n*_ and the resultant short circuit current *I*_*scn*_ as:

5 $${I}_{sc}={I}_{scn}(\frac{E}{{E}_{n}})$$

For a given irradiance intensity, open-circuit voltage *V*_*oc*_ can be given based on a baseline irradiance intensity *E*_*n*_ and the resultant open circuit voltage *V*_*ocn*_ as:

6 $${V}_{oc}={V}_{ocn}+\frac{nkT}{q}\,\mathrm{ln}(\frac{E}{{E}_{n}})$$

Where *n*,* k*, *q* and *T* represent the ideality factor for the photocell, Boltzmann Constant, electron charge and absolute temperature respectively^41^.

In order to generate comparative values with the experimental data, the generalized mathematical model was simplified to a RMP with a circular base as illustrated in Fig. 2(c). Figure 2(d) shows the estimated intensity distribution at the photoactive plane for PD1 embedded inside of cylindrical RMPs (with circular bases) of different diameters. Steps of this simplification are provided in the methods section.

The performance of a soldered PD without RMP or further modifications was considered the baseline (*E*_*n*_, *I*_*scn*_, *V*_*ocn*_) for generating theoretically estimated I_SC_ and V_OC_ values for comparison. The derived model was subsequently validated experimentally to provide a generalised solution for the encapsulation of optical devices within similar micro-pods.

Full details of the mathematical model are provided in supplementary section 2.

### Effect of micro-pod diameter

PD1 soldered onto fine copper wire interconnects were encapsulated with 1.5 mm, 1.9 mm, 2.7 mm, 3.8 mm, 4.8 mm and 5.8 mm diameter RMPs using a clear acrylated urethane resin (Dymax, 9001E-V3.5, Dymax Corporation, Torrington, CT, USA; this resin was used throughout this work unless otherwise specified). Similarly, PD2 soldered onto fine copper interconnects were encapsulated creating RMPs with 2.7 mm, 3.8 mm, 4.8 mm and 5.8 mm outer diameters. The PDs were positioned at the bottom of the resultant RMP (hereafter referred to as the standard PD configuration): Therefore, the depth of positioning also varied with the micro-pod diameter as depicted in supplementary section 3.

**Figure 3:**
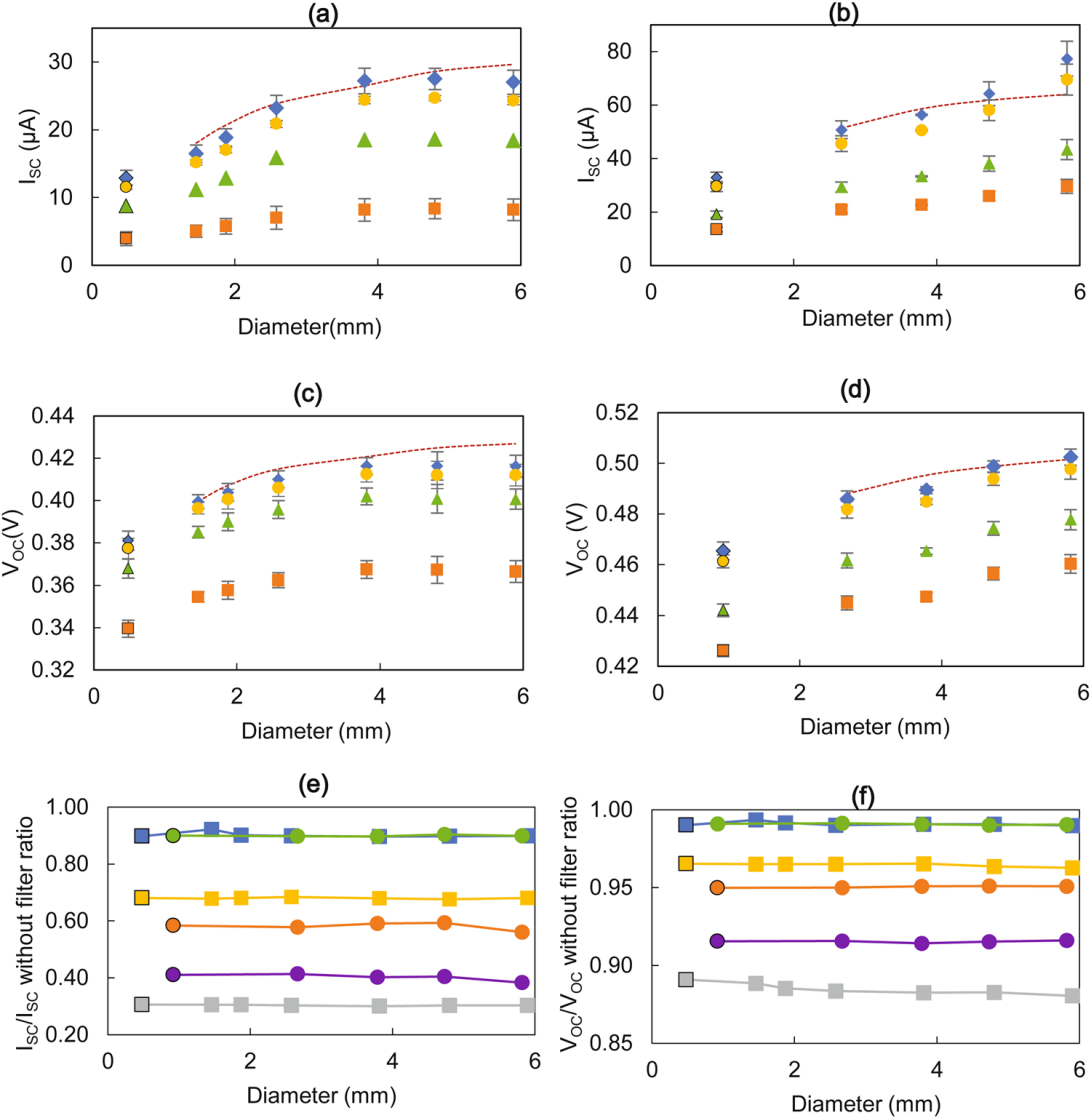
Effect of micro-pod diameter on short-circuit current (I_SC_) and open-circuit voltage (V_OC_) for photodiodes (PDs) embedded in the standard configuration under different optical filters. (**a**) I_SC_ against diameter for TEMD 7000 × 1. (**b**) I_SC_ against diameter for VEMD 6060 × 1. (**c**) V_OC_ against diameter for TEMD 7000 × 1. (**d**) V_OC_ against diameter for VEMD 6060 × 1. Experimental values under no optical filter, 304–785 nm band, 495 nm long pass and 780 nm long pass optical filters are depicted by blue diamonds, orange squares, yellow circles and green triangles respectively. The error bars show the standard deviation from five repeat experiments. The maroon broken lines indicate the estimated values by the mathematical model for the full spectral irradiance (no optical filter). (**e**) The ratio between I_SC_ with and without optical filters against micro-pod diameter. (**f**) The ratio between V_OC_ with and without optical filters against micro-pod diameter. Grey squares, blue squares and yellow squares indicate the measurements under 304–785 nm band optical filter, 495 nm long pass optical filter and 780 nm long pass optical filter respectively for TEMD 7000 × 1. Purple circles, green circles and orange circles indicate the measurements under 304–785 nm band optical filter, 495 nm long pass optical filter and 780 nm long pass optical filter respectively for VEMD 6060 ×1. In all the cases, first data point of each data series (demarcated with a black border) indicate the I_SC_ and V_OC_ values against the width of the photoactive area for non-encapsulated PDs.

The fabricated RMPs were evaluated with the optical test rig under four filter settings; no filter, 485 nm long pass filter, 304–785 nm band pass filter and 780 nm long pass filter with power intensities of 202.22 W/m^2^, 179.60 W/m^2^, 164.04 W/m^2^ and 35.35 W/m^2^ respectively. The I_SC_ and V_OC_ values recorded are illustrated in Fig. 3(a–d), along with a comparison of the values predicted by the mathematical model.

Both the I_SC_ and V_OC_ values for PD1 showed a large increase in values up to a 4 mm diameter, which plateaued afterwards. In the case of PD2, despite the increase in I_SC_ and V_OC_ values, the plateau effect was indistinct for the RMP sizes tested in the experiment.

Figure 3(e,f) show I_SC_ and V_OC_ measurements under three different optical filters relative to the corresponding measurement without an optical filter for PD1 and PD2. These values remained constant for both the PDs before and after encapsulating them inside of RMPs for the range of RMP diameters. This indicated that the spectral band of the light received by the photoactive chip of the PD did not change with the diameter of the RMP. This was an important result as different spectral wavelengths are relevant to different potential applications.

An estimation of I_SC_ and V_OC_ values were calculated using the mathematical model and compared with experimental data points, with a mean average percentage error (MAPE) subsequently calculated. The mathematical model estimated values exhibited a close fit with the experimental data with MAPEs of 7.38% for I_SC_ and 1.40% for V_OC_ for PD1 and 8.13% for I_SC_ and 0.43% for V_OC_ for PD2, considering all RMP sizes. When RMP sizes up to 4.8 mm were considered for PD2, the MAPEs improved to 5.57% for I_SC_ and 0.33% for V_OC_ indicating a better fit of the model. Therefore, it can be concluded that the developed mathematical model was suitable for predicting I_SC_ and V_OC_ values for both the PD types within the given error percentages providing a useful general solution.

### Effect of the depth of positioning the photodiode inside of the resin micro-pod

**Figure 4:**
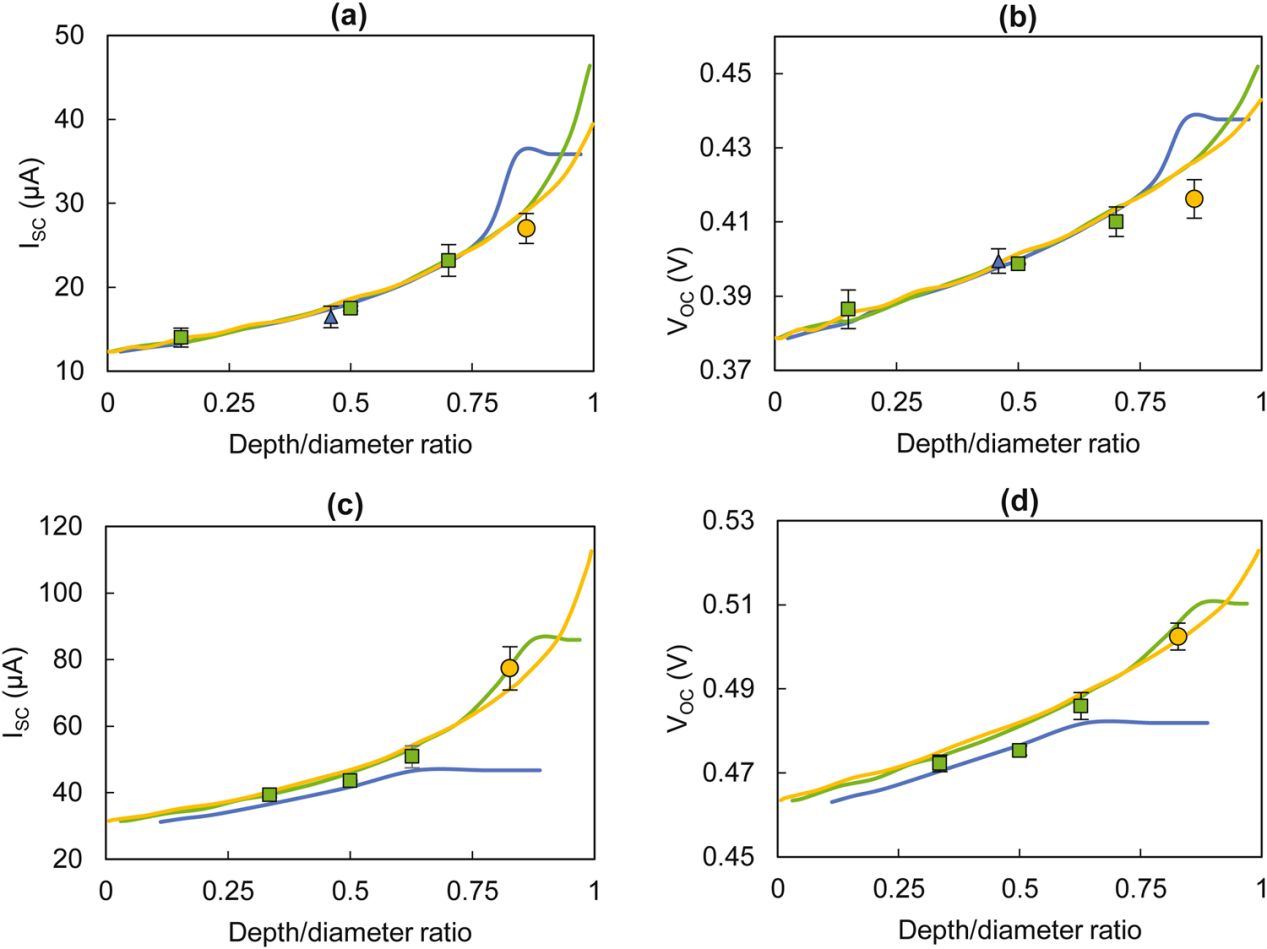
Effect of depth on short-circuit current (I_SC_) and open-circuit voltage (V_OC_) for photodiode embedded resin micro-pods. Experimental (1.5 mm diameter-blue triangles, 2.7 mm diameter-green squares, 5.8 mm diameter- yellow circles) and mathematical model estimated (1.5 mm diameter-blue lines, 2.7 mm diameter-green lines, 5.8 mm diameter-yellow lines) values given for (**a**) I_SC_ against depth/diameter ratio for TEMD 7000 × 1, (**b**) V_OC_ against depth/diameter ratio for TEMD 7000 × 1, (**c**) I_SC_ against depth/diameter ratio for VEMD 6060 × 1 and (**d**) V_OC_ against depth/diameter ratio for VEMD 6060 × 1. Measurements conducted using the optical test rig without optical filters. The error bars show the standard deviation from five repeat experiments.

PDs were embedded inside of 2.7 mm diameter RMPs at three depth levels (the standard PD configuration, at the extreme top, and at the centre). Microscopic images of the RMPs confirmed that the level of accuracy of the PDs positioning inside RMPs to be within ~±5% (detailed in supplementary section 3). The experimental results for I_SC_ and V_OC_ are presented against the depth to RMP diameter ratio (hereafter referred to as DDR) in Fig. 4 along with predicted values from the mathematical model for three different RMP diameters (1.5 mm, 2.7 mm, and 5.8 mm).

The results confirmed that depth was a key determining factor of the opto-electronic parameters. The mathematical model predicted values showed a good fit with the experimental data with a MAPE of 7.41% and 6.25% for I_SC_ and 1.36% and 0.80% for V_OC_ for PD1 and PD2 respectively. This further confirmed the utility of the mathematical model for accurate predictions of I_SC_ and V_OC_ values.

Based on the predicted values, it was clear that the PD diameter did not have a direct effect on the I_SC_ and V_OC_ up to ~0.75 DDR for PD1. The model predicted that for PD1, a 1.5 mm diameter RMP would yield maximum I_SC_ and V_OC_ at around ~0.85 DDR. In the case of PD2, the 2.7 mm diameter RMPs generated maximum values for I_SC_ and V_OC_, at around a DDR of 0.87. For PD2, it was not possible to fabricate 1.5 mm RMPs since the SMD packaging of the PD2 was 2 mm in width. Nevertheless, the mathematical model predicted that the I_SC_ and V_OC_ values for the photoactive chip (~0.9 mm wide) of the PD2 embedded inside the 1.5 mm RMP, reached maximum values at around a DDR of 0.65. These values were significantly lower than corresponding maximum values of PD2 embedded within 2.7 mm and 5.8 mm diameter RMPs.

These experimental results along with the mathematical model estimations indicated that DDR was a key parameter when optimising opto-electronic characteristic: DDR showed a greater direct influence on the PD performance than the absolute values of depth or the diameter of the micro-pods. In general, higher DDRs yielded higher values for I_SC_ and V_OC_. Nevertheless, in certain cases there existed peak I_SC_ and V_OC_ points beyond which a marginal decrease in I_SC_ and V_OC_ was observed. This was attributed to the diversion of rays away from the photoactive area of the PD and an increase in the path length (leading to higher light absorption) of the light inside the RMP. As mentioned previously, in practical scenarios, it may not be possible to achieve DDRs that yield the theoretical maximum I_SC_ /V_OC_ value due to the thickness and width of the PD.

When the results for PD1 and PD2 were compared it was clear that the RMP diameters which realised the highest I_SC_ /V_OC_ values were different for each PD type. This was an indication that the width of the photoactive plane was also a key determining factor in selecting the size of the RMP. The RMP should be large enough to accommodate the photoactive device and there may exist a practical maximum RMP diameter value owing to design constrains governed by the end applications and process parameters. It is important to examine how the I_SC_ and V_OC_ values behave within these limits to determine the most suitable RMP diameter.

### Effect of the micro-pod material

The effects of the material properties of elements in optical systems are well understood. They can define how electromagnetic waves reflect, refract and get absorbed, for an optical element with a given geometry.

**Figure 5:**
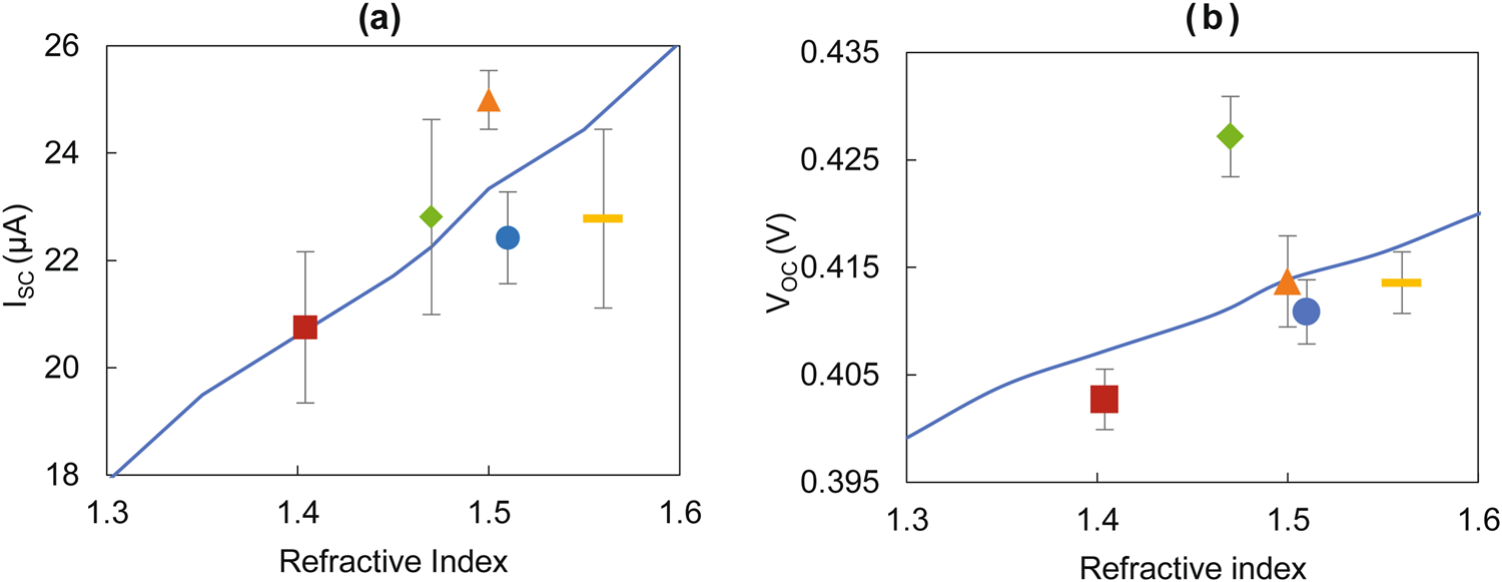
Effect of resin material type on short-circuit current (I_SC_) and open-circuit voltage (V_OC_) for photodiode embedded resin micro-pods. Experimental and mathematical model estimated values given for TEMD 7000 × 1 embedded inside 2.7 mm diameter micro-pod in the standard configuration. (**a**) I_SC_ against refractive index. (**b**) V_OC_ against refractive index. Blue circle, orange triangle, green diamond, maroon square and yellow rectangle represent the resin types 9001E-V3.5, OP29, OPT4200, OPT 7020 and OPT 5200 respectively. The blue line indicates the mathematical model predicted values with decadic attenuation coefficient of 0.0001 db/mm. Measurements conducted using the optical test rig without optical filters. The error bars show the standard deviation from five repeat experiments.

Experiments were conducted using five different types of resins (including the standard resin) by embedding PD1 type within RMPs of 2.7 mm diameter (in the standard PD configuration). These resins had refractive indices ranging from 1.404 to 1.56. As the resultant RMPs exhibited different levels of shrinkages after UV curing, experimental results were normalized based on the diameter of the RMP with standard resin to indicate comparable I_SC_ and V_OC_ values. The results were plotted against the refractive indices of material types as depicted in Fig. 5. In addition, the mathematical model estimated I_SC_ and V_OC_ values were indicated in the same figures.

The results indicated an overall increase in I_SC_ and V_OC_ values with the increase in refractive index. The silicone based resin OPT 7020 yielded the lowest I_SC_ while the optical grade acrylated urethane resin OP29 exhibited the highest I_SC_ values.

It is noteworthy that for the RMP geometries considered in this study, the refractive index had a significantly higher impact than the attenuation coefficient on the irradiance intensity measured at the photoactive plane. The refractive index governs the ray concentrating effects, while also defining the reflective losses (at material boundaries), which are significant in magnitude. As an example, in the case of a 2.7 mm diameter RMP made of the standard resin (1.51 refractive index and 0.0001 dB/mm attenuation coefficient), theoretical values for the percentage of light reflected at the surface of the resin micro-pod and the percentage absorbed by the RMP were ~4% and less than 0.05% respectively. The model-estimated values for each data point were calculated based on the assumption that all of the resins absorbed light in a similar way, which may not have been the case. However detailed information was not forthcoming in the literature^42^. When compared with the experimental data, the mathematical model exhibited a MAPE of 6.03% and 1.44% for I_SC_ and V_OC_ values respectively, which indicated a good fit.

### Photodiode embedded textile yarns

PD embedded textile yarns with PD1 and PD2 type devices were realized by encapsulating them inside of 2.7 mm diameter RMPs in the standard PD configuration and finally integrating them within a fibrous sheath (2.7 mm was the smallest RMP size that could be employed for both PD types).

**Figure 6:**
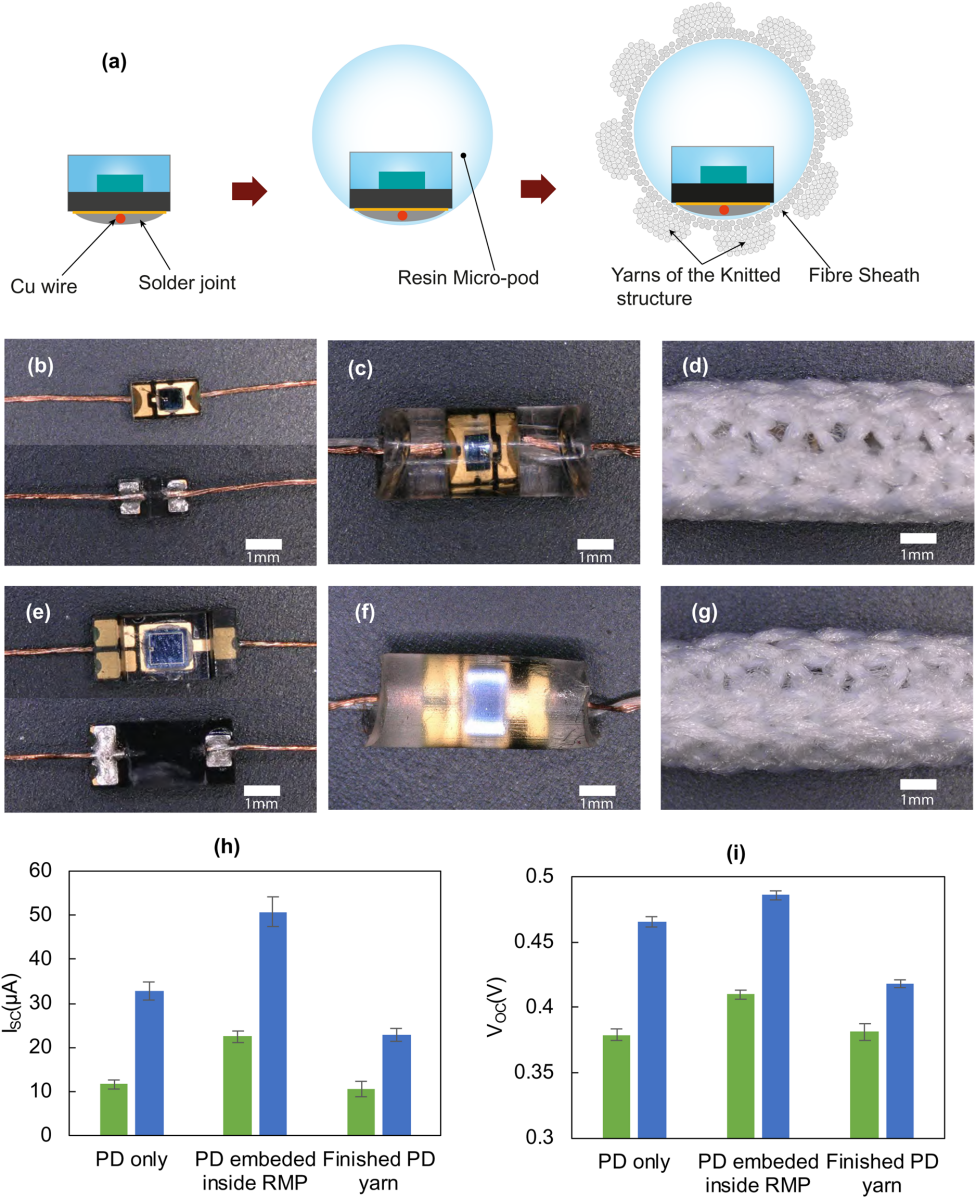
Appearances and change in opto-electronic properties at different stages of the photodiode (PD) yarn fabrication process. (**a**) Schematic cross sectional view of PD devices, top view images of. (**b**–**d**) TEMD 7000 × 1 type and (**e**–**g**) VEMD 6060 × 1 type PD devices after soldering copper wires, after embedding inside micro-pods and in the completed yarn form respectively. (**h**,**i**) Comparison of experimental values of TEMD 7000 × 1 (green bars) and VEMD 6060 × 1 (blue bars) for before encapsulation, after embedding inside resin micro-pods of 2.7 mm diameter (standard resin in the standard PD configuration), and for the completed PD yarn (with the same RMP, sheathing fibres and knitted structure) respectively. Measurements conducted using the optical test rig without optical filters. The error bars show the standard deviation from five repeat experiments.

The structure of the PD embedded textile yarn and images of the devices after each step in the fabrication process are given in Fig. 6(a–g). The finalized PD embedded textile yarn had a maximum outer diameter of ~4.4 mm.

It was evident that the sheathing fibres and knit-braid structure had a negative effect of the I_SC_ and V_OC_, due to the reduction of light flux on to the RMP caused by scattering^43^ and absorption^44^ of light by the fibres (see comparative results in Fig. 6(h,i)). Nevertheless, the finished yarn generated I_SC_ and V_OC_ values comparable to the original PDs.

### Individual and combined effect of the components

**Figure 7:**
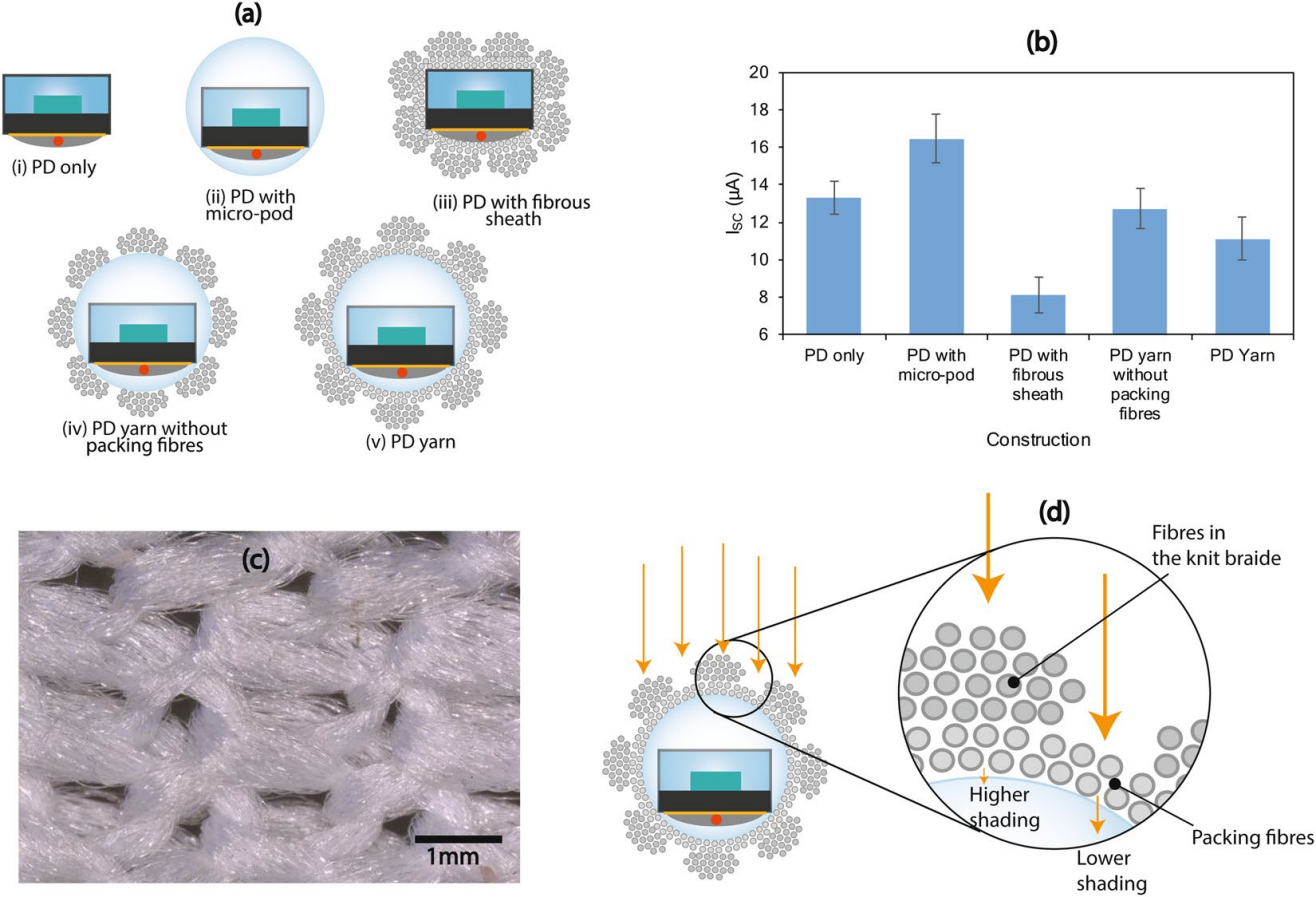
Individual effect of different components of the PD embedded yarn on the short circuit current (I_SC_). **(a)** Schematic cross sectional view of different yarn constructions with PD1. **(b)** The I_SC_ values for each yarn construction under full spectral incident light using the optical test rig, **(c)** Microscopic image of the knit braid structure. **(d)** Schematic of depicting the transmission of light through the packing fibres and knit braid structure in the fibrous sheath.

To understand the individual effects of the resin micro-pod, packing fibres and the knit-braided structure on the performance of the PD-embedded E-yarn, a series of PD1 embedded strands with different constructions were fabricated and I_SC_ measurements were conducted, as shown in Fig. 7 below. These yarns had an RMP of 1.5 mm (with the PD in standard PD configuration) and final yarn thickness of ~2 mm. A 1.5 mm RMD was used for these experiments as this was determined to be the optimal size for PD1.

As discussed previously, the light concentrating effects of the RMP were evident with a 23% increase in I_SC_ for PD with the RMP (Fig. 7a(ii)) compared to PD only (Fig. 7a(i)). This was reduced by around 48% after covering the RMP with the knit-braided fibrous sheath (Fig. 7a(v)). When the E-yarn was made without packing fibres (Fig. 7a(iv)) the I_SC_ values were ~29% lower than the PD with the RMP values. When the E-yarn was made without a micro-pod (Fig. 7a(iii); the soldered PD was manually inserted into a knit-braid structure), the I_SC_ values were 39% lower than for the PD only (Fig. 7a(i)) value, which showed the combined shading effect of the packing fibres and the knit-braid on the PD.

These results provide a clear indication of the individual and combined effects of the RMP, packing fibres and the knit-braid structure on the amount of light transmitted to the photosensitive area of the PD. The knit-braid (linear density of the knit-braid ~385 mg/m) showed a higher shading effect than the packing fibres (linear density of packing fibres ~70 mg/m), due to the higher fibre density created by the loop structure of the knit.

The shading effect of the composite fibrous sheath can be attributed to the amount of light scattered and absorbed (attenuated) by the fibres in the sheath. The knit-braid has a semi-open tubular structure (Fig. 7c) where a proportion of the light can be directly transferred to the interior (packing fibre layer) of the PD-yarn through the openings without scattering or absorption. The degree of openness of a knitted structure is defined by the porosity^45^, which is dependent on the thickness of the yarns and loop structure of the knitted structure. A proportion of light that is received by the yarns in the knit-braid is partially reflected or scattered at the surface of the fibres. Each individual fibre will scatter a fraction of incident light depending on the refractive index of the fibre material. Therefore, the total amount of light transmitted through the knit-braided sheath and packing fibres will decay exponentially with fibre density. Polyester fibres typically have a refractive index of ~1.54^46^ meaning that 4–5% of the incident light is reflected by a single fibre.

Light penetrating into the fibre is absorbed by the polymer and delustrants^46^ present within the fibre. The light absorption is significantly lower than the light scattering especially for the fine and white/light colour fibres employed in this study. Due to these phenomena, only a proportion of the incident light will be transferred though a bundle of fibres to the inner layer of the yarn. The same effect, with a lower magnitude, is given by the packing fibres since its fibre density is smaller than the knit-braid.

### Wash durability testing

The wash durability of PD1 embedded yarns were tested in both E-yarn form and fabric form. The E-yarn form experiments, as described in the methods section, subjected the E-yarns to high mechanical agitation with machine washing and tumble-drying. In the second method, a woven structure was created by inserting PD1 embedded E-yarns as the weft during the fabric manufacturing process, and was subjected to machine-washing (inside a wash bag) and line drying which was a representation of domestic applications.

**Figure 8:**
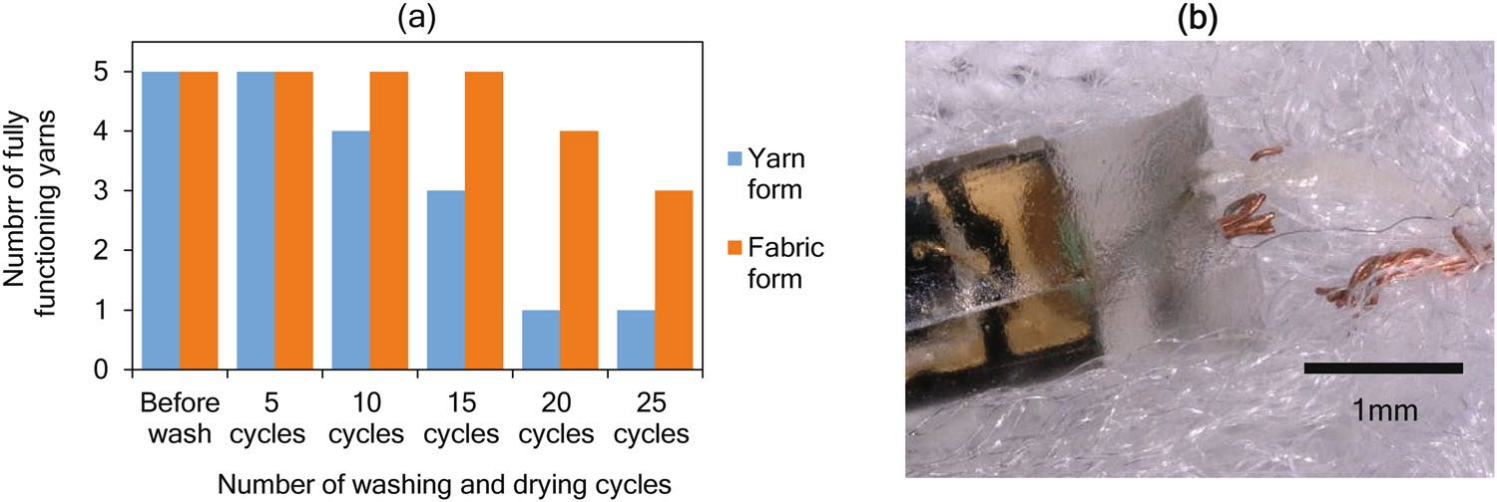
Wash durability testing. **(a)** Wash durability results of PD embedded E-yarns tested in yarn form and fabric form. **(b)** Microscopic image of a dissected PD embedded E-yarn with copper wire breakage at RMP after wash.

The wash test results (Fig. 8a) confirmed the durability of the PD embedded E-yarns under multiple washing and drying cycles. In the E-yarn form experiments, where machine washing and tumble-drying was used, the first failure was observed between five to ten washes, while the first failure of the fabric form samples occurred between 15 to 20 washes. Out of the five samples 20% survived 25 cycles in for the E-yarn form experiments, whereas 60% remained fully functional after 25 cycles for the fabric form experiments. The E-yarns tested in fabric form survived longer due to the structural support given by the woven fabric structure, wash bag, and the exposure to lower degree of mechanical stresses. The failed E-yarns were dissected to investigate the cause of failure. Microscopic images confirmed that all of the failures were due to breakage of the copper wires (Fig. 8b) close to the RMP, possible caused by fatigue from bending and twisting of the PD embedded E-yarn close to the RMP. No physical deterioration of the RMP was observed on any of the failed PD embeded E-yarns.

### Conclusion

The fabrication of photodiode (PD) embedded E-yarns was successfully demonstrated for the first time. The PDs were embedded inside of cylindrical resin micro-pods (RMPs) made of a clear polymeric resin, and then covered by a fibrous sheath consisting packing fibres and a tubular knitted structure made of polyester fibres. The effects of the resin micro-pod geometry and material on the opto-electronic properties (short circuit current and open circuit voltage) were investigated in detail by evaluating experimental data along with a mathematical model. The experimental data exhibited a good fit with the mathematical model, which proved the utility of the model in establishing design rules for the PD E-yarns within given constraints. The results indicated that for the two PD types discussed in this work, the depth (represented as a ratio of the diameter of the micro-pod) at which the photoactive plane of the PD is positioned inside the RMP is a key factor in determining the I_SC_ and V_OC_ values. The experiments conducted with micro-pods made of a series of resin types with varying refractive indices showed the positive effect that higher refractive indexes had on I_SC_ and V_OC_.

The results of the experiments conducted with the PD embedded E-yarns revealed that the I_SC_ and V_OC_ values of the finished yarns were comparable to the values produced by bare PDs, and lower than the values of PDs embedded inside the micro-pods. The knit-braid of the fibrous sheath was the main reason for this reduction, while the packing fibres also contributed. In general, I_SC_ was highly sensitive to the variations of the geometry and material type of the yarn components (due to the linear relationship between I_SC_ and light intensity), while V_OC_ values varied modestly with similar variations (due to the logarithmic relationship between V_OC_ and light intensity). The wash testing confirmed the durability of PD embedded E-yarns when woven into a fabric under multiple cycles of washing and drying, which is critical for the adoption of these yarns for many wearable applications.

With the demonstration of a PD embedded E-yarn using this technique, there is no doubt that the integration of various types of optical sensors inside of textile yarns is a viable proposition. Further, the development of a generalised theoretical model allows for the creation of optimised E-yarn designs for other miniaturized opto-electronic devices and textile based optical sensing.

## Methods

### Sample production

#### Making electrical interconnects between the solder-pads and copper wires

The step-by-step process of attaching the PDs to copper wires is illustrated in supplementary section 4. First, the PD was placed on a black pigmented silicone base with the two solder pads facing upwards. A seven-strand copper wire (Knight Wire, Potters Bar, UK; linear density = 120 mg/m, single strand diameter = 50 µm) was placed on top of the two solder pads and held under tension. Approximately 3 µl of lead free solder paste (SolderPlus® S965D500A6, Nordson EFD, Dunstable, UK) was dispensed onto the copper wire at the solder pad using a pneumatic dispensing system (EFD Ultimus II dispenser system, Nordson EFD, Dunstable, UK) and reflow soldered using an IR spot reflow soldering system (PDR IR-E3 Rework System, PDR- Design & Manufacturing Centre, Crawley, UK).

The copper wire length between the solder pads was removed by cutting the copper wires at the inner edges of the solder pads.

#### Encapsulating the soldered PDs inside of RMPs

The apparatus for encapsulation (see supplementary section 5) consisted of one rotary and one stationary fibre clamp and two cymbal tensioners before and after the two fibre clamps. This allowed the soldered photodiode to be securely positioned inside of a PTFE tube (Adtech Polymer Engineering Ltd., Gloucester, UK) which was mounted on the XYZ adjustable platform.

The soldered PD-copper wire strand was reinforced by using a 100 denier Vectran yarn (Vectran™, Kuraray America Inc., Houston, TX, USA) guided through the PTFE tube. The PD position inside the PTFE tube was adjusted using the rotary clamp and XYZ movable platform to achieve the intended orientation of the PD inside the resultant micro-pod.

Once positioning was complete a predetermined volume of resin was injected into the spaces between the PD-copper strand, Vectran filaments, and the inner walls of the PTFE tube (as an example, with PD1 and a 2.7 mm diameter tube ~20 µL of resin was used). Typically, the resin volume was controlled to create a cylindrical micro-pod with a length 2–3 mm longer than the length of the PD (1.0–1.5 mm longer on each side). Then the resin was cured using a UV source (BlueWave^TM^ 50, Dymax Corporation, Torrington, CT, USA) for 60 seconds. Finally, the copper strand was released from the clamps and cymbal tensioners, before forcing the cured micro-pod out of the PTFE tube by applying a tensile force on the Vectran yarn.

#### Embedding the micro-pod inside a knit-braid fibre sheath

Small diameter circular warp-knitting machines (RIUS MC-Knit braiders with 2.0 and 4.0 mm inner diameter hollow cylinders with six and eight needles, outer diameters of the hollow needle cylinders were 10.0 mm; RIUS, Barcelona, Spain) were used to form the fibrous sheath around the micro-pod and copper/Vectran strand. The first set of PE packing fibres were delivered straight through the inside of the hollow needle cylinder with the RMP, copper interconnects and Vectran fibre, thus these did not form loops. The second set of PE yarns (48 f/167 dtex) were delivered to the knitting needles on the outer surface of the needle cylinder, which formed the warp knitted structure (knit-braid structure) around the packing fibres, creating the final PD embedded E-yarn. The packing fibres were used to retain the RMS and copper interconnects in the centre of the E-yarn.

For 2.7 mm diameter RMPs an eight needle hollow cylinder with a 4.0 mm inner diameter was selected for the knit-braid, and eight PE yarns (48 f/167dtex) were used for packing fibres. Eight PE yarns were used by the needles to form the knit-braid. For 1.5 mm diameter RMPs a six needle hollow cylinder with a 2.0 mm inner diameter was selected, and six PE yarns (48 f/167dtex) were used to form the knit-braid. Four PE yarns were used for packing fibres.

### Simplification of the generalized mathematical model

The generalized mathematical model was simplified to a RMP with circular bases as illustrated in Fig. 2(c), where the diameter of the cylinder was *R*. The centre of the cylinder in the circular plane was taken as the origin (0,0) and the boundary surface of the RMP was given by Eq. (7).

7 $$y=g(x)=\sqrt{({R}^{2}-{x}^{2})}\,$$

The incident beam of light was normal to the photoactive plane therefore γ = 0. Based on the defined circular geometry.

8 $$\theta ={\sin }^{-1}(\frac{x}{R})$$

9 $$\beta ={\sin }^{-1}\{\frac{\sqrt{({R}^{2}-{x}^{2})({{n}_{r}}^{2}{R}^{2}-{x}^{2})}+{x}^{2}}{{n}_{r}{R}^{2}}\}$$

Eqs (7–9) are used to specify the Eqs (1–6) to arrive at derivations for I_SC_ and V_OC_ values for a PD embedded within a micro-pod with circular cross section.

The complete derivation of the mathematical model is provided in the supplementary section 4.

### Evaluating the optoelectronic behaviour of the PDs, PD micro-pods and PD yarns

#### Optical Test Rig

A customized optical test rig was developed to generate a reliable and reproducible light source with a suitable spectral and irradiance output to conduct I_SC_ and V_OC_ measurements. The test rig also provided a means of using light filters, and a mechanism to position the specimens in a repeatable manner.

The test rig consisted of a quarts tungsten halogen lamp (QTH10(/M), Thorlabs Inc., Ely, UK) coupled with a glass diffuser and a convex lens with a 50 mm diameter circular beam output. The diameter of the output from the lamp was reduced to 25 mm to match the size of the light filters that were subsequently aligned to the light beam using an adjustable filter holder. A frame with a removable sample holder was mounted next to the filter holder. The backside of the sample holder was attached onto a plastic sheet on which the test sample was attached. The PD test samples were mounted using electrical insulation tape in a way that the PD sample was positioned at the centre of the incident beam of light with the photosensitive side directly facing the lamp. The copper interconnects of the PD yarn samples were connected to a high precision digital multi-meter (Model 34410 A 6 ½, Agilent Technologies LDA UK Limited, Stockport, UK). Each test sample was conditioned inside the test rig for about 30 seconds before reading measurements from the multi-meter.

To maintain a consistent temperature (~25 °C) of the test samples, a Peltier cooler (Supercool® PE-161-12-15, Gothenburg, Sweden) and thermocouple based feedback controlled temperature control system was also employed. A schematic and an image of the optical test rig are given in the supplementary section 6.

#### Wash durability testing

The wash durability testing for PD embedded E-yarns were conducted, based on the procedure 4N outlined in the British standard BS EN ISO 6330:2012; Textiles — Domestic washing and drying procedures for textile testing^47^.

The first set of tests were conducted in E-yarn form (attached to cotton T-shirts by zig-zag stitching) and were both machine washed and tumble-dried. The second set of samples were woven into fabrics and machine washed inside a wash bag before being line dried. All samples were tested before washing and after 5, 10, 15, 20, 25 wash and dry cycles for their electrical performance using the optical test rig. Full details of the sample preparation and test conditions are provided in supplementary section 7.

## Electronic supplementary material

Supplementery information
