# Supplementary material for: Photodiodes embedded within electronic textiles
Source: Sci Rep. 2018 Nov 1;8:16205. doi: 10.1038/s41598-018-34483-8 (PMC6212443; doi:10.1038/s41598-018-34483-8)
Supplement: Supplementary file 1 — Supplementery information [file 41598_2018_34483_MOESM1_ESM.pdf]

Supplementary Information

Photodiodes embedded within electronic textiles

Achala Satharasinghe[1], Theodore Hughes-Riley[1]* & Tilak Dias[1]

[1] Advanced Textiles Research Group, School of Art & Design, Nottingham Trent University, Bonington Building, Dryden Street, Nottingham NG1 4GG, UK. Correspondence should be addressed to A.S (achala.satharasinghe2016@my.ntu.ac.uk or T.H.R (theodore.hugheriley@ntu.ac.uk)

Section 1: Detailed specifications, IV curves, and variation analysis of the photodiodes used in the experiment.

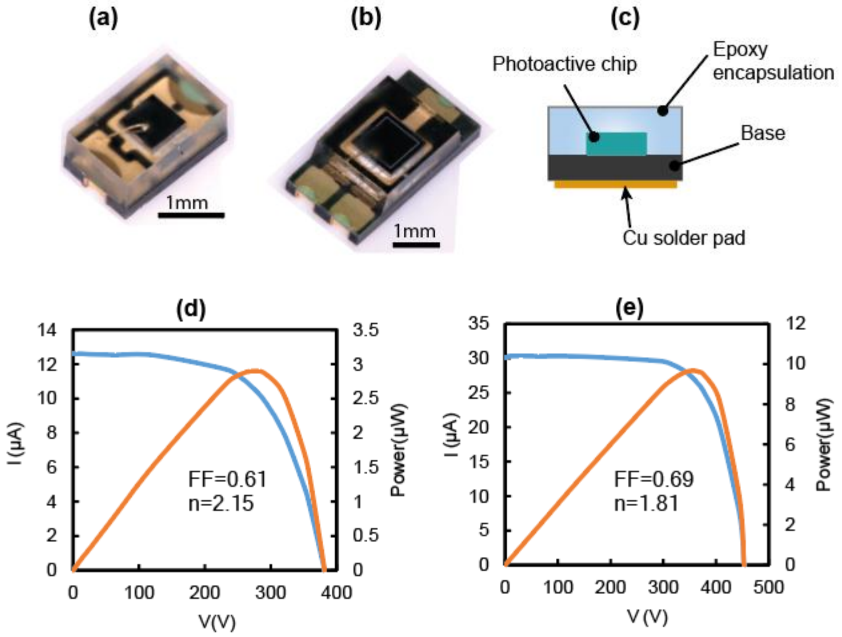

Figure S1 -Two photodiode (PD) types employed in the experiments (**a**) Image of the TEMD 7000x01 PD (**b**) Image of the VEMD 6060x01 PD (**c**) Front view schematic of the PDs (**d**) Characteristic curves, fill factor (FF) and ideality factor (n) for TEMD 7000x01 PD (**e**) Characteristic curves, fill factor (FF) and ideality factor (n) for VEMD 6060x01 PD . (IV curves given in blue lines and power curves given in orange lines).

Fig. S1(a-c) show images and a cross-sectional depiction of the two photodiodes tested. Fig. S1 (d,e) depict typical characteristic curves (IV and power) for the two PD types, generated under the baseline test settings, along with the corresponding fill factors (FF) of and ideality factors (n), estimated for PD1 and PD2 respectively. PD2 had a larger area to perimeter ratio, and showed a ~13% higher fill factor and lower ideality factor compared to PD1, possibly due to lower edge recombination of charge carriers.

Table S1 and S2 provide full specifications of the PDs.

Table S1 – TEMD 7000x01 photodiode basic characteristics

**BASIC CHARACTERISTICS** (T_{amb} = 25 °C, unless otherwise specified)						
**PARAMETER**	**TEST CONDITION**	**SYMBOL**	**MIN.**	**TYP.**	**MAX.**	**UNIT**
Forward voltage	I_F = 50 mA	V_F		1		V
Breakdown voltage	I_R = 100 μA, E = 0	$V_{(BR)}$	60			V
Reverse dark current	V_R = 10 V, E = 0	I_{ro}		1	3	nA
Diode capacitance	V_R = 0 V, f = 1 MHz, E = 0	C_D		4		pF
	V_R = 5 V, f = 1 MHz, E = 0	C_D		1.3		pF
Open circuit voltage	E_e = 1 mW/cm², λ = 950 nm	V_o		350		mV
Temperature coefficient of V_o	E_e = 1 mW/cm², λ = 950 nm	TK_{Vo}		-2.6		mV/K
Short circuit current	E_e = 1 mW/cm², λ = 950 nm	I_k		3		μA
Temperature coefficient of I_k	E_e = 1 mW/cm², λ = 950 nm	TK_{Ik}		0.1		%/K
Reverse light current	E_e = 1 mW/cm², λ = 950 nm, V_R = 5 V	I_{ra}	2.4	3	3.6	μA
Angle of half sensitivity		φ		± 60		deg
Wavelength of peak sensitivity		$λ_p$		900		nm
Range of spectral bandwidth		$λ_{0.1}$		350 to 1120		nm
Rise time	V_R = 10 V, R_L = 1 kΩ, λ = 820 nm	t_r		100		ns
Fall time	V_R = 10 V, R_L = 1 kΩ, λ = 820 nm	t_f		100		ns

Table S2- VEMD 6060x01 basic characteristics

**BASIC CHARACTERISTICS** (T_{amb} = 25 °C, unless otherwise specified)						
**PARAMETER**	**TEST CONDITION**	**SYMBOL**	**MIN.**	**TYP.**	**MAX.**	**UNIT**
Forward voltage	I_F = 50 mA	V_F	-	0.85	1.1	V
Breakdown voltage	I_R = 100 μA, E = 0	$V_{(BR)}$	20	-	-	V
Reverse dark current	V_R = 10 V, E = 0	I_{ro}	-	0.03	5	nA
Diode capacitance	V_R = 0 V, f = 1 MHz, E = 0	C_D	-	11	-	pF
	V_R = 5 V, f = 1 MHz, E = 0	C_D	-	4.8	-	pF
Open circuit voltage	E_e = 1 mW/cm^2, λ = 950 nm	V_o	-	360	-	mV
Temperature coefficient of V_o	E_e = 1 mW/cm^2, λ = 950 nm	TK_{Vo}	-	-3.1	-	mV/K
Short circuit current	E_e = 1 mW/cm^2, λ = 950 nm	I_k	-	5	-	μA
Temperature coefficient of I_k	E_e = 1 mW/cm^2, λ = 835 nm	TK_{Ik}	-	0.1	-	%/K
Reverse light current	E_e = 1 mW/cm^2, λ = 950 nm, V_R = 5 V	I_{ra}	3.5	5	6.5	μA
	E_e = 1 mW/cm^2, λ = 890 nm, V_R = 5 V	I_{ra}	-	7	-	μA
Angle of half sensitivity		φ	-	± 70	-	deg
Wavelength of peak sensitivity		λ_p	-	820	-	nm
Range of spectral bandwidth		$\lambda_{0.1}$	-	380 to 1070	-	nm
Rise time	V_R = 10 V, R_L = 50 Ω, λ = 830 nm	t_r	-	60	-	ns
Fall time	V_R = 10 V, R_L = 50 Ω, λ = 830 nm	t_f	-	50	-	ns

Variation analysis for TEMD 7000x01 before and after encapsulating inside resin micro-pods.

Thirty samples created using PD1 were tested under identical conditions (202.22 W/m^2 with no light filter; hence referred to as the baseline test setting) using the optical test rig, with I_{SC} and V_{OC} recorded. The soldered PDs showed an average, standard deviation (SD) and co-efficient of variation (CV) of 12.87μA, 1.14 and 8.86% for I_{SC} (Fig. S2.a) and 0.381V, 0.0045 and 1.18% for V_{OC} values (Fig. S2.b).

Twenty of the thirty soldered PDs were randomly selected and encapsulated inside cylindrical RMPs with ~2 .7mm diameter using the Dymax 9001E-V3.5 acrylated urethane resin. The PDs were encapsulated at the bottom of the resultant RMP. The encapsulated samples had average, SD and CV values of 23.20 μA, 1.88 and 8.12 % for I_{SC} (Fig. S2.a) and 0.4101 V, 0.0040 and 0.97 % for V_{OC} (Fig. S2.b) values. From these results, it was clear that there was a significant increase in I_{SC} and V_{OC} values after the encapsulation. There existed a variation in I_{SC} values before the encapsulation process, which remained unaltered after the encapsulation, indicating that no measurable variation was introduced to the I_{SC} values by the encapsulation process. These variations were within the manufacturer's specifications.

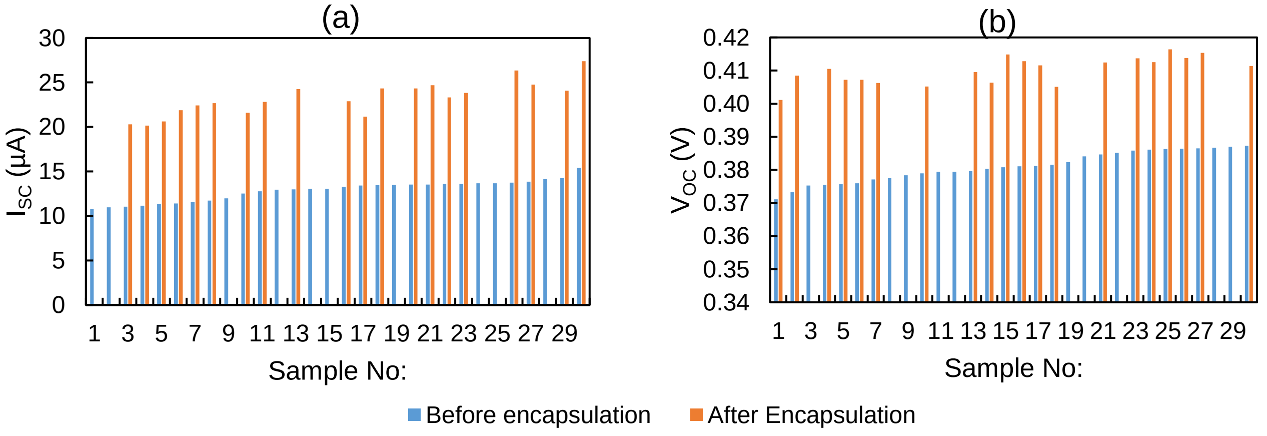

Figure S2 - Variation analysis of TEMD 7000x01 Photodiode before and after encapsulation inside 2.7mm micro-pods in standard PD configuration. (**a**) Short circuit current. (**b**) Open circuit voltage.

Section 2: Complete derivation of the mathematical model

In order to understand and characterise the effects observed for the encapsulated PDs a generalized ray tracing mathematical model, which predict the light intensity within the RMP, was proposed.

Generalized Ray Tracing Model

In its simplest terms, a single ray incident of light can be considered. The ray had an intensity E_i and angle γ to the vertical axis, which met the boundary of the cylindrical micro-pod defined by $y = g(x)$ at co-ordinates x_0, y_0, as illustrated in Fig. S3. A fraction of the incident ray was reflected (E_r) at the boundary surface and the remaining fraction (E_p) was refracted into the RMP. The refracted ray was attenuated during its travel inside the RMP before reaching the plane of measurement (E_t). A fraction of the ray (E_{rc}) was partially reflected at the photocell surface, and the residual ray (E_c) was transmitted to the semiconductor.

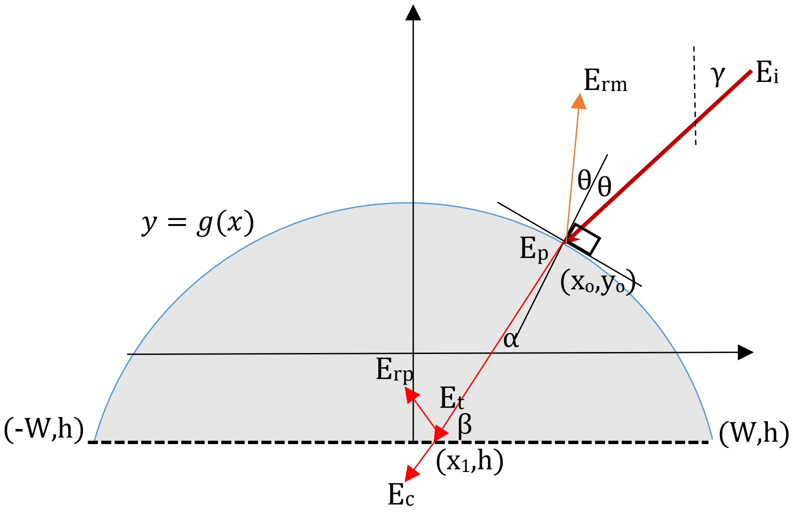

Figure S3 - Generalized ray-tracing model depicting the cross-sectional view of the cylindrical micro-pod.

E_i- Intensity of incident ray
E_{rm}- Intensity of reflected ray at air micro-pod boundary
E_{rp}- Intensity of reflected ray at the plane of measurements
E_p-Intensity of the ray penetrated through the micro-pod
E_t -Intensity of transmitted ray at the plane of measurement
E_c -Intensity of transmitted to the photocell
γ- Angle of incident light to the vertical axis
g(x) -Boundary surface function of the material
n_r - Refractive index of the resin material relative to air
n_p - Refractive index of the photocell material relative to air
μ - decadic attenuation coefficient of the material
θ - Angle of incident light to the normal of the boundary surface
α - Angle of refracted light to the normal of the boundary surface
β - Angle of transmitted light to the horizontal axis

$$E_p = E_i - E_{rm} \qquad \text{(I)}$$

Based on the geometry:

$$\alpha + \beta + \gamma - \theta = \frac{\pi}{2}$$

$$\left(\frac{dy}{dx}\right) = -\cot(\beta + \alpha)$$

$$x_1 = x_0 - \left\lfloor \frac{g(x_o) - h}{\tan\beta} \right\rfloor \qquad \text{(II)}$$

From Snell's law of refraction:

$$n_r * \sin\alpha = \sin\theta$$

From Fresnel equation for partial reflection of non-polarized light at the boundaries of non-magnetic material:

$$\frac{E_{rm}}{E_i} = \left(\frac{1}{2}\right) * \left\{\left[\frac{\cos\theta - n_r\sqrt{1-(\sin\theta/n_r)^2}}{\cos\theta + n\sqrt{1-(\sin\theta/n_r)^2}}\right]^2 + \left[\frac{n_r\cos\theta - \sqrt{1-(\sin\theta/n_r)^2}}{n_r\cos\theta + \sqrt{1-(\sin\theta/n_r)^2}}\right]^2\right\} \qquad \text{(III)}$$

Based on the theory of absorption of electromagnetic radiation inside a homogeneous material:

$$E_t = E_p * 10^{(-\mu l)} \qquad \text{(IV)}$$

Where: $l = [g(x_o) - h] \operatorname{cosec}\beta$

Using the Fresnel equation for partial reflection of non-polarized light at the boundaries of non-magnetic material, the reflection at the micropod-photocell boundary can be given as:

$$\frac{E_{rp}}{E_t} = \left(\frac{1}{2}\right) * \left\{\left[\frac{n_r\sin\beta - n_p\sqrt{1-(\frac{n_r}{n_p}\cos\beta)^2}}{n_r\sin\beta + n_p\sqrt{1-(\frac{n_r}{n_p}\cos\beta)^2}}\right]^2 + \left[\frac{n_p\sin\beta - n_r\sqrt{1-(\frac{n_r}{n_p}\cos\beta)^2}}{n_p\sin\beta + n_r\sqrt{1-(\frac{n_r}{n_p}\cos\beta)^2}}\right]^2\right\} \qquad \text{(V)}$$

The intensity of the ray transmitted to the photocell:

$$E_c = E_t - E_{rp}$$

The average Intensity between two points (x_a, h) and (x_b, h) on the horizontal plane along (x_1,h) are given by:

$$E_{AVG} = \left(\int_{x_a}^{x_b} E_c . dx/(x_b - x_a)\right) \qquad \text{(VI)}$$

Simplification of the generalized model for a circular cross section

In order to generate comparative values with the experimental data, the generalized mathematical model was simplified to a RMP with a circular bases.

Incident light is uniform and parallel to the vertical axis. The boundary surface is circular and the width of the section is equal to the diameter.

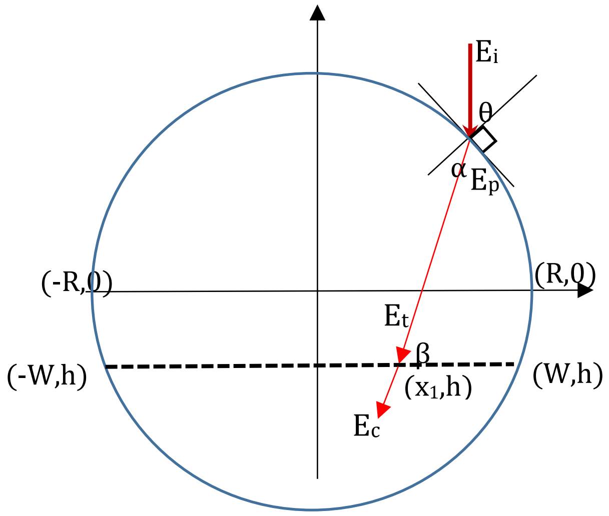

$$y = g(x) = \sqrt{(R^2 - x^2)}$$

y=0

$$\theta + \beta - \alpha = \frac{\pi}{2}$$

$$\left(\frac{dy}{dx}\right) = -\tan\theta$$

$$\left(\frac{dy}{dx}\right) = -\frac{x}{\sqrt{(R^2 - x^2)}}$$

Here $-R < h < R$

Figure S4 - Simplified ray tracing model for a circular cross section.

$\tan\theta = \frac{x}{\sqrt{(R^2-x^2)}} \Rightarrow \sin\theta = \frac{x}{R}, \cos\theta = (\frac{\sqrt{(R^2-x^2)}}{R})$ and

$$n_r * \sin\alpha = \sin\theta \Rightarrow \sin\alpha = (\frac{x}{n_r R}), \cos\alpha = \frac{\sqrt{(n_r{}^2R^2-x^2)}}{nR}$$

$$\sin\beta = \sin\left(\frac{\pi}{2} - \theta + \alpha\right) = \cos(\alpha - \theta) = \frac{\sqrt{(R^2-x^2)(n_r{}^2R^2-x^2)}+x^2}{nR^2}$$

$$\cos\beta = \cos\left(\frac{\pi}{2} - \theta + \alpha\right) = \sin(\theta - \alpha) = x(\sqrt{(n_r{}^2R^2 - x^2)} - \sqrt{(R^2 - x^2)})/(R^2 * n_r)$$

$$\tan\beta = \frac{\sqrt{(R^2 - x^2)(n_r{}^2R^2 - x^2)} + x^2}{x\left(\sqrt{(n_r{}^2R^2 - x^2)} - \sqrt{(R^2 - x^2)}\right)}$$

$$\Rightarrow x_1 = x_0 - \left[\frac{g(x_o)-h}{\tan\beta}\right] = x_0\left[1 - \left(\sqrt{(R^2 - x_o{}^2)} - h\right) * \frac{\left(\sqrt{(n_r{}^2R^2-x_o^2)} - \sqrt{(R^2-x_o^2)}\right)}{\sqrt{(R^2-x_o^2)(n_r{}^2R^2-x_o^2)}+x_o^2}\right] \quad \text{(VII)}$$

$$\Rightarrow l = [g(x_o) + h]\csc\beta = \frac{R^2 n_r\left(\sqrt{(R^2 - x^2)} - h\right)}{\sqrt{(R^2 - x^2)(n_r{}^2R^2 - x^2)} + x^2}$$

$$\frac{E_{rm}}{E_i} = \left(\frac{\left(1-n_r{}^2\right)^2}{2}\right) * \left\{\left[\frac{R}{\sqrt{(n_r{}^2R^2-x^2)}+\sqrt{(R^2-x^2)}}\right]^4 + \left[\frac{n^2(R^2-x^2)-x^2}{\left(\sqrt{(n_r{}^2R^2-x^2)}+n^2\sqrt{(R^2-x^2)}\right)^2}\right]^2\right\} \quad \text{(VIII)}$$

Estimating short circuit current and open circuit voltage for a crystalline photocell encapsulated inside of a RMP

The intensity estimates were converted into estimated I_{SC} and V_{OC} values using the fundamentals of semiconductor photovoltaics. For a crystalline silicon photocell embedded inside a RMP, with a rectangular photo-active area and photoactive width matching the measurement plane, and measurement width discussed in the above ray tracing model, the irradiance intensity on the photocell E can be given as the:

$$E = E_{AVG}$$

Based on the theory the relationships between short-circuit current (I_{sc})and irradiance intensity E for a photocell can be given as:

$$I_{sc} = K_E * E$$

Where K_E is a constant that characterizes the relative variation of short circuit current as a function of irradiance intensity.

The nominal values for short circuit current (I_{scn}) and open circuit voltage (V_{ocn}) of the photocell are determined experimentally under nominal irradiance intensity (E_n) before encapsulating inside a RMP.

Using the above equations the short circuit current I_{sc} for an irradiance intensity E can be estimated as:

$$I_{sc} = I_{scn} * \left(\frac{E}{E_n}\right) \quad \text{(IX)}$$

Also the open circuit voltage V_{oc} for a given irradiance intensity E can be given as:

$$V_{oc} = V_{ocn} + \frac{nkT}{q} * \ln\left(\frac{E}{E_n}\right) \quad \text{(X)}$$

Here k - Boltzmann Constant

n - Ideality factor

q - Electron charge

T- Absolute temperature

The ideality factor (n) for the photocell is determined using the IV curve of the photocell under nominal irradiance condition E_n. The IV curve is fitted to an exponential function in the below form:

$$I = \alpha - \beta e^{\gamma V}$$

Where I is the current V is the voltage and α, β and γ are constants. According to theory the ideality factor is given by:

$$n = \frac{q}{\gamma k T}$$

Comparison of optical behaviour of actual and model defined photodiode

When the effects of optical reflection, refraction and absorption were considered, it was estimated that the epoxy layer (on the un-encapsulated PD) made an insignificant difference on the irradiance intensity at the photoactive plane for all the scenarios evaluated with these particular PDs.

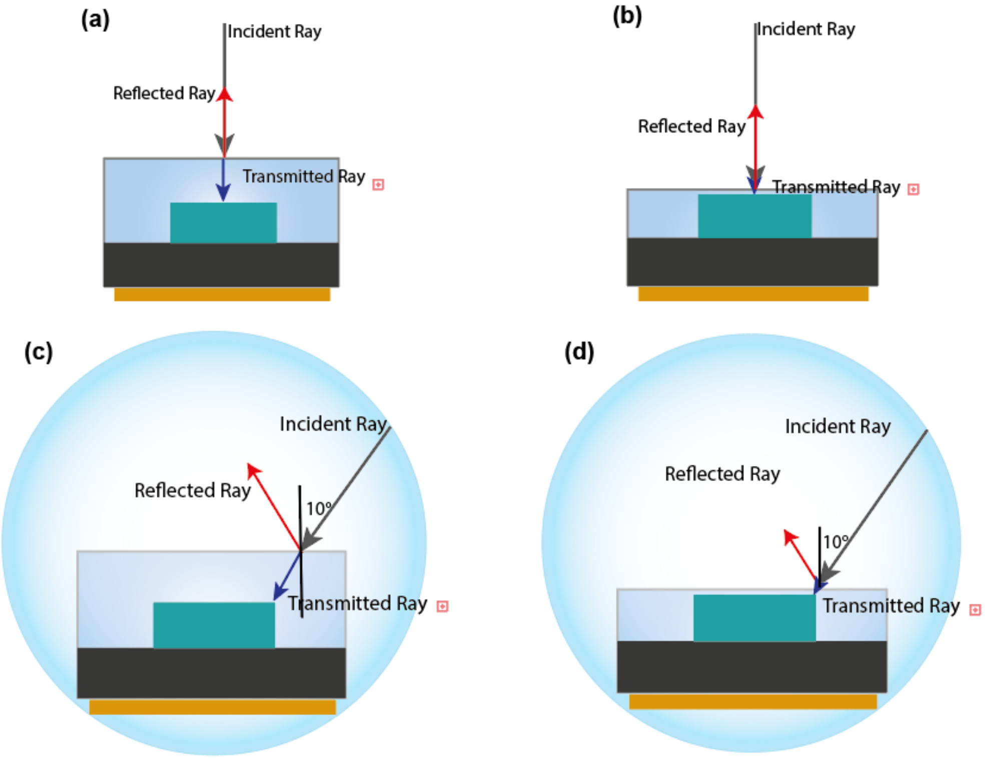

Figure S5 - Comparison of actual and mathematical model defined Photodiode (PD) configuration. (**a**) Actual PD before encapsulation, (**b**) Model defined PD before encapsulation. (**c**) Actual PD after encapsulation. (**d**) Model defined PD after encapsulation.

Before encapsulation

When the actual PD and model defined PD configuration was compared before encapsulation, the loss in transmission due to Fresnel reflection was the same due to the same acrylic-epoxy material interfaces. The drop in intensity due to absorption of light by the epoxy material for actual PD configuration is given as:

$$\%\ optical\ absorption\ by\ the\ epoxy\ layer\ with\ 0.5mm\ thickness\ = \left(1 - 10^{-(.5x0.0001)}\right) * 100$$
$$= 0.0115\%$$

It is clear that the model-defined configuration has only a fraction of the above loss due to substantially small thickness of the epoxy layer.

After encapsulation

Let’s consider a ray which passes the air-acrylic resin interface, enters the acrylic material and reaches an edge of the photoactive material. Based on the calculations in this case the ray reaches the acrylic material at an angle no more than 10° to the vertical axis, for all the scenarios discussed in this paper for both PD types. Both the rays undergo the same material interfaces therefore; the reduction in transmittance due to Fresnel reflectance (partial reflectance at material interfaces) is the same. Since the epoxy layer in the mathematically modelled configuration is very thin, the effect of refraction can be neglected.

The from the Snell’s law the angle of transmitted ray into the epoxy material is given by θ as below.

$$1.51 * Sin(10^o) = 1.55 * Sin\theta$$

$$\theta = 9.74^o$$

The deflection (d) of the incident ray due to refraction in the actual PD configuration can be calculated as below for an epoxy layer thickness of 0.5mm. The actual epoxy layer thickness of PDs are less than that.

$$d = 0.5 * (tan10^o - tan9.74^o)$$

$$d = 0.00234mm$$

This deflection is negligible compared to the width (0.92mm) of the photoactive material (less than 0.25%). Additionally, similar attenuation coefficients of the acrylic and epoxy materials result in a negligible difference in loss due to optical absorption.

Based on the above calculations, it can be concluded that the effect on light intensity due to the physical differences between the actual PD configuration and mathematical model is negligible.

Section 3 - Depth of encapsulation of photodiodes for different micro-pod diameters in different configurations; theoretical and practical limits and experimental results.

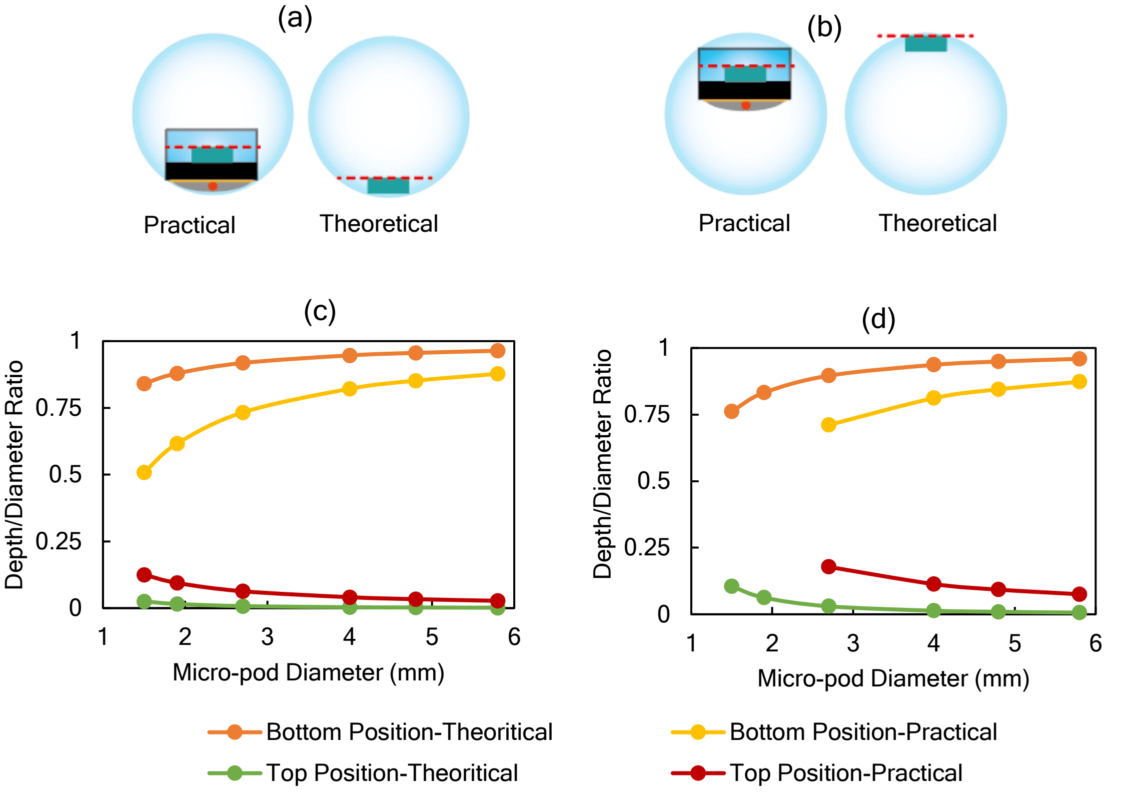

Figure S6 - Theoretical and practical the depths of positioning the photoactive planes of photodiodes inside of the resin micro-pods (**a**) Schematic of the PD in the bottom position. (**b**) Schematic of PD in the top position. (**c, d**) Depth to diameter ratio for top and bottom positions against micro-pod diameter for: (**c**) TEMD 7000x01 (**d**) VEMD 6060x01

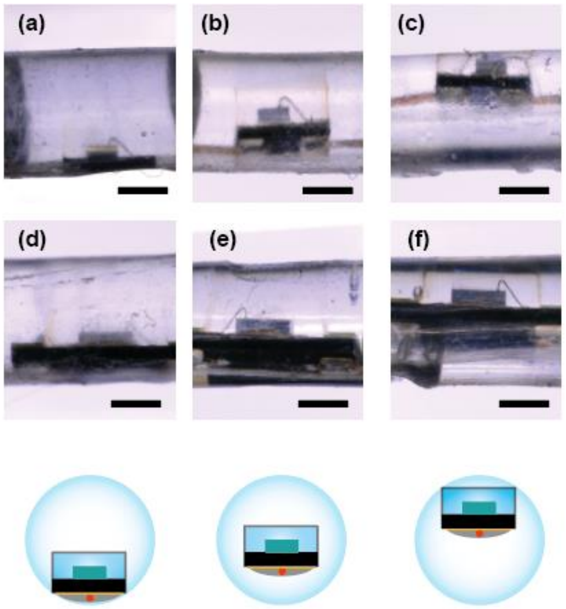

Figure S7 - Images of positioning of the photodiodes inside of 2.7mm diameter resin micro-pods. (**a**) TEMD7000x01 embedded at the bottom position. (**b**) TEMD7000x01 embedded at centre position. (**c**) TEMD7000x01 embedded at top position. (**d**) VEMD6060x01 embedded at the bottom position, (**e**) VEMD6060x01 embedded at the centre position. (**f**) VEMD6060x01 embedded at top position. (**bottom**) Schematics of the three PD positions.

Section 4 - Process of soldering copper wire onto photodiodes

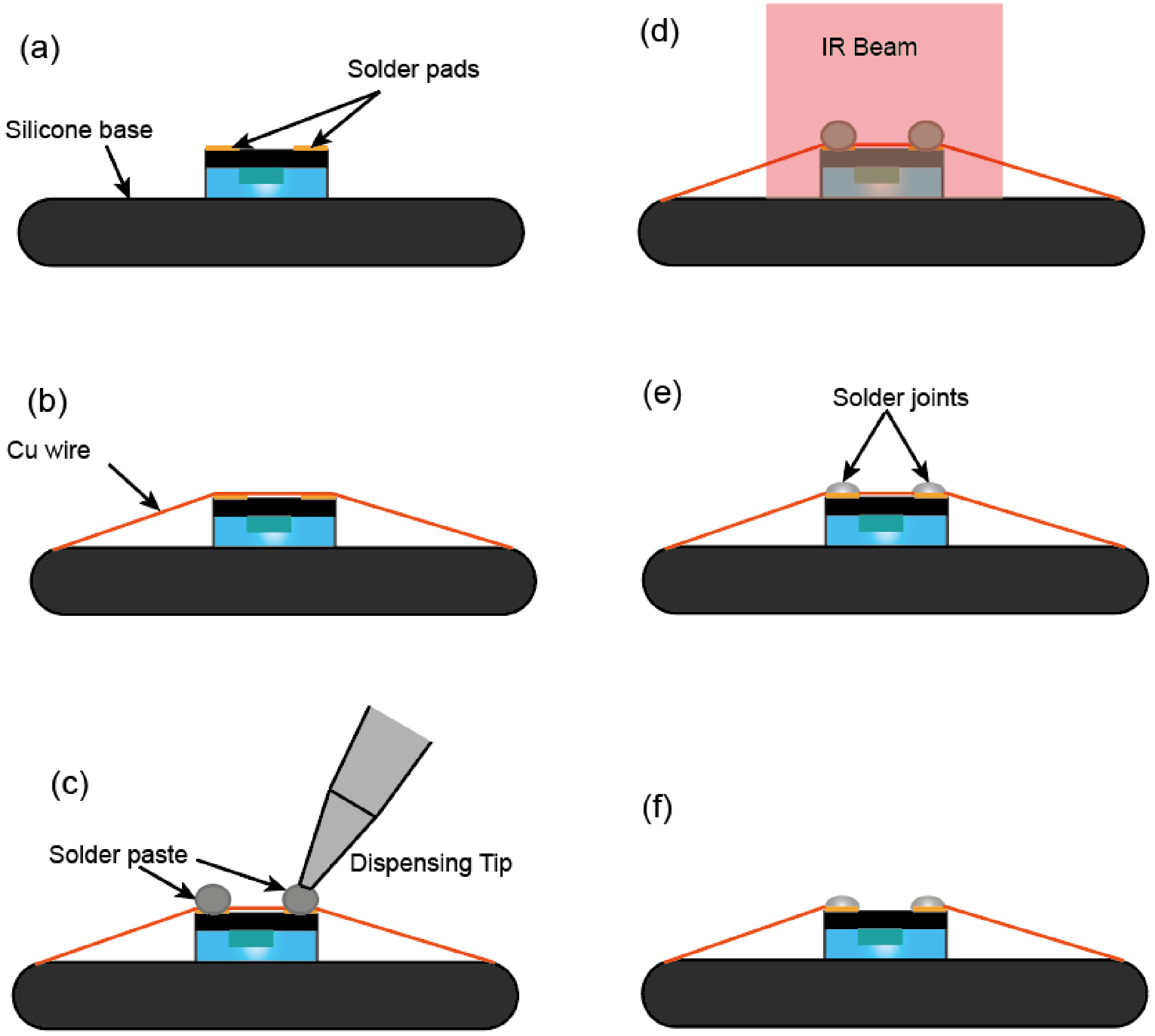

Figure S8 - Soldering process for photodiodes (PDs). (**a**) The PD placed on the silicone base with solder pads upwards. (**b**) Copper wire placed over the solder pads of the PD. (**c**) Solder paste dispensed onto the solder pads and Cu wire. (**d**) Solder pads and solder paste exposed to IR beam. (**e**) Solder joints formed. (**f**) Copper wire length between solder joints removed.

Section 5 – Apparatus for positioning and encapsulating photodiodes inside resin micro-pods

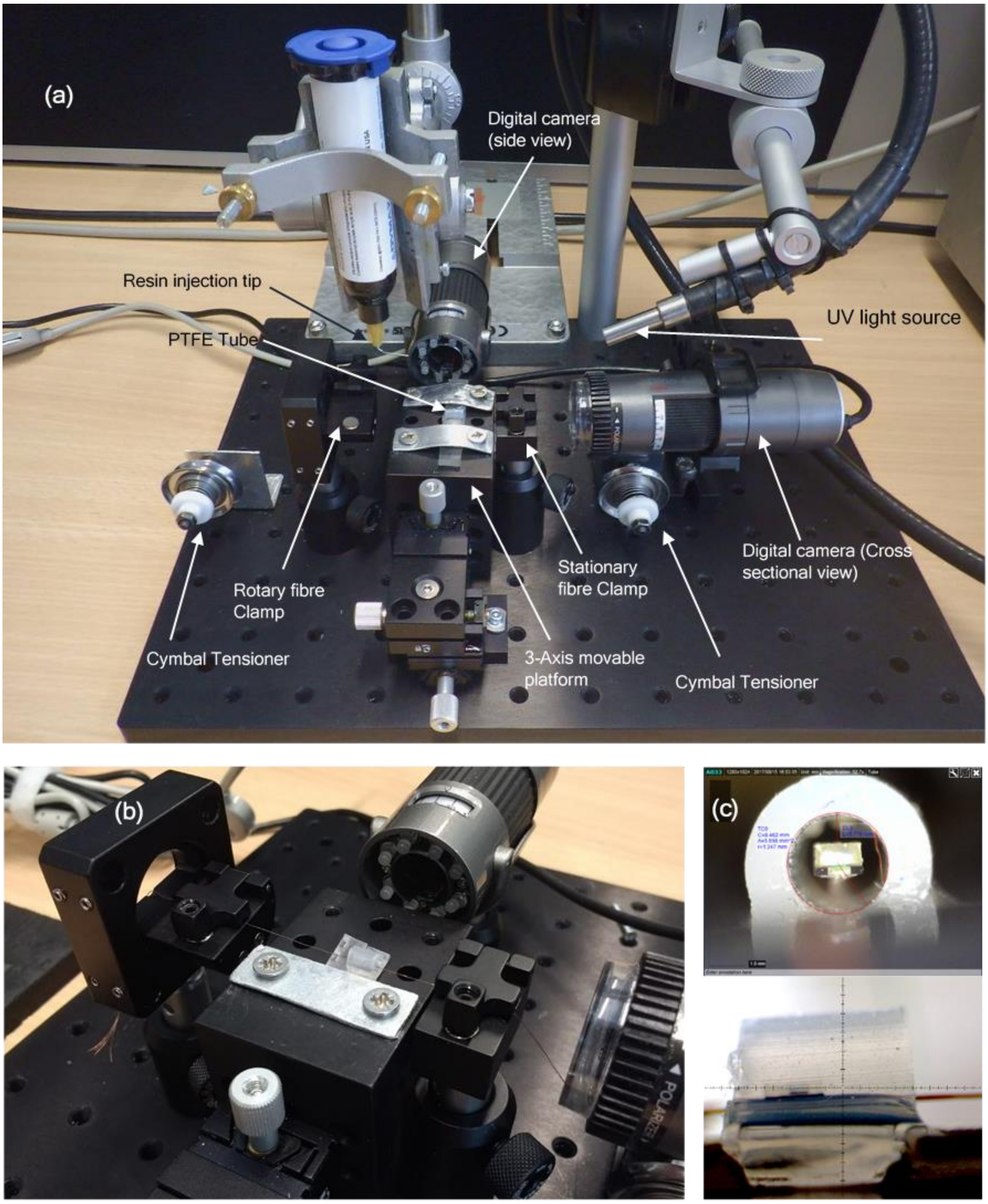

Figure S9 – Apparatus for positioning and encapsulating photodiodes inside of the resin micro-pods. (**a**) Full view of the test rig. (**b**) A soldered PD inserted through the PTFE tube encapsulation mold. (**c**) Images of a positioned photodiode taken from the digital cameras.

Section 6 : Optical test rig and light source, optical filters employed for testing photodiode devices

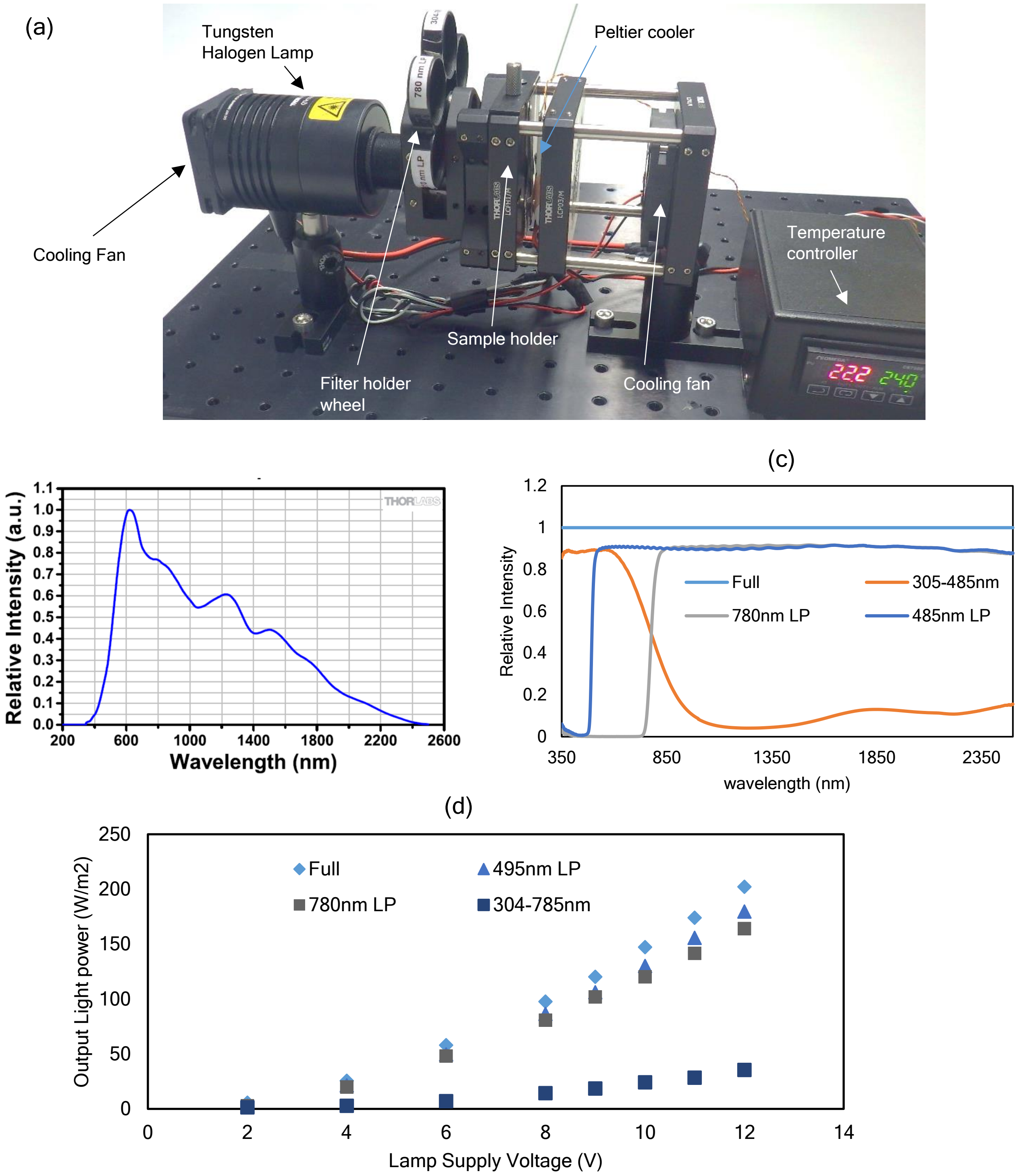

Figure S10 - Optical test rig and light source settings. (**a**) Image of the optical test rig. (**b**) Manufacturers specification for the emission spectrum of the tungsten halogen lamp (Courtesy of Thorlabs Inc.). (**c**) Transmission spectra for different filters employed. (**d**) Relationship between supply voltage and optical output of the lamp through different light filters.

Section 7- Wash durability testing

The wash durability tests were conducted, according to procedure 4N outlined in the British standard BS EN ISO 6330:2012; Textiles — Domestic washing and drying procedures for textile testing. All samples were evaluated before washing, and after 5, 10,15, 20 and 25 wash and dry cycles for their performance.

In the first method, the test specimens were prepared by attaching the PD1 embedded yarns (1.5mm RMP, 2mm yarn diameter) to onto cotton fabric swatches using a zig-zag embroidery stitch (See Fig. S11(a)), with the photoactive side of the yarn fully exposed. Each cotton fabric swatch was attached with five electronic yarns and was affixed onto white cotton T-shirt using male/female press-stud snap attachments as depicted in Fig. S11(b).

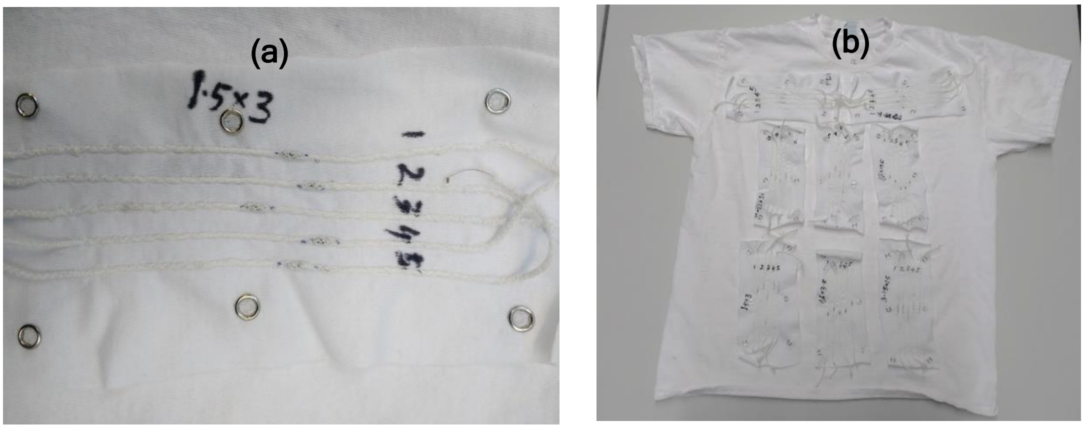

Figure S11 – Sample preparation for wash durability testing in yarn form. (**a**) PD yarns attached to cotton fabric swatches using zigzag stitching. (**b**) Fabric swatches with PD yarns attached cotton T-shirt using metallic snap fastener press studs.

Table S3- Wash and tumble dry test conditions as directed by the BS EN ISO 6330:2012 standard and actually set for the test.

	**Parameter**	**Procedure 4N in BS EN ISO 6330:2012**	**Settings selected on the machine**
**Washing**	Temperature	40±3 °C	40 °C
	Washing time	15 minutes	15 minutes
	Rinsing time	10 minutes	10 minutes
	Spinning time	5 minutes	6 minutes
	Spinning RPM	800±20	800
**Tumble drying**	Temperature, RPM and Time	Drying time selected to achieve 2(±3)% final humidity.	Drying program set to Sportswear (1 hour 47 minutes)

A front loading type programmable domestic washing machine (Boshch Logixx 8 VarioPerfect, BSH Home Appliances Ltd, Milton Keynes, United Kingdom) and a front loading condenser type domestic tumble-drying machine (Boshch Classixx 8, BSH Home Appliances Ltd, Milton Keynes, United Kingdom) were employed for the testing. Wash and tumble dry programmes with approximately similar settings to the wash and tumble dry cycles prescribed in the wash standard were selected as mentioned in the Table S3.

The second wash test was conducted on PD1 embedded yarn in woven fabric form (Fig. S12(a)). The fabric was constructed with a basket weave structure. Cotton yarns were used as the warp and PD embedded yarns were inserted as the weft with the photoactive side of the PD yarn fully exposed. Between two adjacent PD embedded yarns, five knit braided yarns without a core were inserted along with cotton weft yarns in an alternating order as shown in Fig. S12(b). In this case the fabrics were machine washed (similar to the previous case) inside a washbag and line-dried under room temperature.

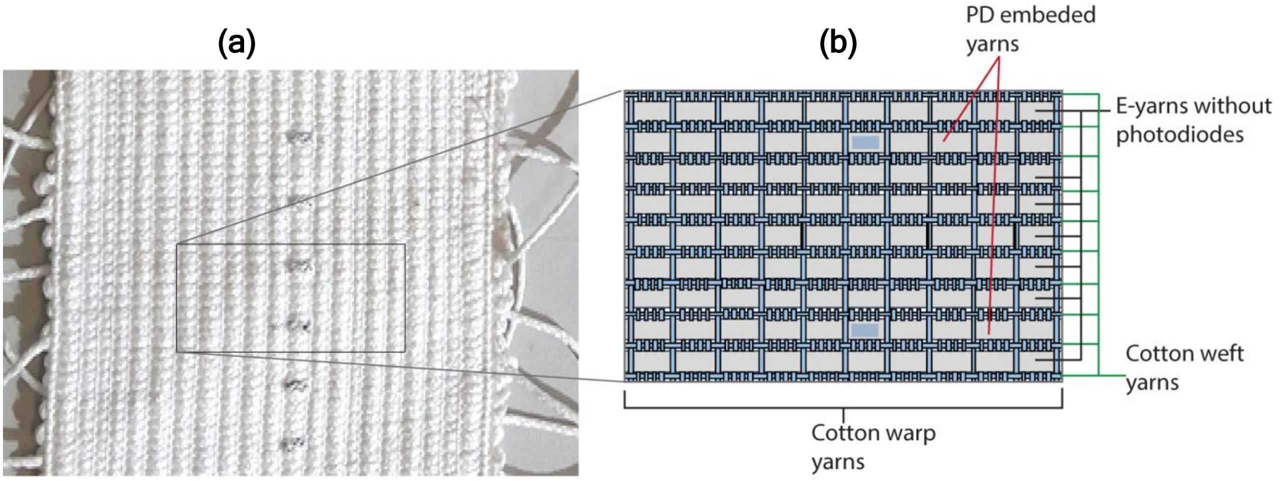

Figure S12 – Sample preparation for wash durability testing in fabric form. (**a**) Picture of the woven fabric developed with PD yarns inserted in warp direction. (**b**) Schematic illustration of the woven structure of the fabric
